# Potato StRWA2 recruits the E3 ligase StSNIPER2 to destabilize NLRs and promote *Phytophthora infestans* infection

**DOI:** 10.1093/hr/uhag072

**Published:** 2026-03-03

**Authors:** Zhengyu Chen, Ziwei He, Juan Du, Yuhe Li, Siqi Niu, Aifang Ma, Qian Chen, Hailong Guo, Jun Fan, Maozhi Ren, Guangyuan Xu, Daolong Dou, Jinguang Yang, Maofeng Jing, Xiaodan Wang

**Affiliations:** State Key Laboratory of Agricultural and Forestry Biosecurity, MOA Key Lab of Pest Monitoring and Green Management, College of Plant Protection, China Agricultural University, Beijing 100193, China; State Key Laboratory of Agricultural and Forestry Biosecurity, MOA Key Lab of Pest Monitoring and Green Management, College of Plant Protection, China Agricultural University, Beijing 100193, China; State Key Laboratory of Agricultural and Forestry Biosecurity, MOA Key Lab of Pest Monitoring and Green Management, College of Plant Protection, China Agricultural University, Beijing 100193, China; State Key Laboratory of Agricultural and Forestry Biosecurity, MOA Key Lab of Pest Monitoring and Green Management, College of Plant Protection, China Agricultural University, Beijing 100193, China; State Key Laboratory of Agricultural and Forestry Biosecurity, College of Plant Protection, Nanjing Agricultural University, Nanjing 210095, China; College of Biological Sciences, China Agricultural University, Beijing 100193, China; State Key Laboratory of Agricultural and Forestry Biosecurity, MOA Key Lab of Pest Monitoring and Green Management, College of Plant Protection, China Agricultural University, Beijing 100193, China; State Key Laboratory of Agricultural and Forestry Biosecurity, MOA Key Lab of Pest Monitoring and Green Management, College of Plant Protection, China Agricultural University, Beijing 100193, China; State Key Laboratory of Agricultural and Forestry Biosecurity, MOA Key Lab of Pest Monitoring and Green Management, College of Plant Protection, China Agricultural University, Beijing 100193, China; Institute of Urban Agriculture, Chinese Academy of Agricultural Sciences, Chengdu National Agricultural Science and Technology Center, Chengdu 610000, China; State Key Laboratory of Agricultural and Forestry Biosecurity, MOA Key Lab of Pest Monitoring and Green Management, College of Plant Protection, China Agricultural University, Beijing 100193, China; State Key Laboratory of Agricultural and Forestry Biosecurity, MOA Key Lab of Pest Monitoring and Green Management, College of Plant Protection, China Agricultural University, Beijing 100193, China; State Key Laboratory of Agricultural and Forestry Biosecurity, College of Plant Protection, Nanjing Agricultural University, Nanjing 210095, China; Key Laboratory of Tobacco Pest Monitoring Controlling & Integrated Management, Tobacco Research Institute of Chinese Academy of Agricultural Sciences, Qingdao 266101, China; State Key Laboratory of Agricultural and Forestry Biosecurity, College of Plant Protection, Nanjing Agricultural University, Nanjing 210095, China; State Key Laboratory of Agricultural and Forestry Biosecurity, MOA Key Lab of Pest Monitoring and Green Management, College of Plant Protection, China Agricultural University, Beijing 100193, China

## Abstract

Nucleotide-binding leucine-rich repeat receptors (NLRs) are central to plant immunity, yet the mechanisms regulating their homeostasis remain poorly understood. In this study, we identify StRWA2 as a susceptibility factor in potato (*Solanum tuberosum*) that negatively regulates NLR-mediated resistance to *Phytophthora infestans*. StRWA2 destabilizes NLR proteins R3a and Rpi-blb2 via the 26S proteasome, suppressing NLR-mediated hypersensitive responses (HR). Mechanistically, StRWA2 recruits the E3 ubiquitin ligase StSNIPER2 (SNC1-INFLUENCING PLANT E3 LIGASE REVERSE 2) and enhances its E3 ligase activity, enabling StSNIPER2-dependent ubiquitination and degradation of NLRs. Furthermore, we confirm the necessity of this partnership, where silencing *NbSNIPER2a/b* reduced StRWA2-mediated plant susceptibility, while expression of a ligase-dead StSNIPER2 variant (StSNIPER2 ^H123Y^) restored NLR stability and plant resistance. Crucially, we obtained *StRWA2*-silenced potato plants via the RNA interference (RNAi), which conferred resistance to *P. infestans* with no observable growth penalties compared to wild-type controls. Together, this study identified a susceptibility factor RWA2 from potato that recruits the E3 ligase SNIPER2 to destabilize NLRs. Our findings reveal a critical NLR regulation mode and propose RWA2 as a promising target for engineering disease resistance in crops.

## Introduction

During plant evolution, plants have developed a multifaceted innate immune system to protect themselves. Pattern-triggered immunity (PTI) is activated by the recognition of pathogen-associated molecular patterns (PAMPs) through host pattern recognition receptors (PRRs), triggering a basal immune response. In contrast, effector-triggered immunity (ETI) is activated by the recognition of specific pathogen effector proteins by plant resistance (R) proteins, typically triggering hypersensitive response (HR) [1–3]. PTI and ETI act synergistically to confer robust and broad-spectrum disease resistance [4, 5]. The majority of resistance (*R*) genes encode nucleotide-binding leucine-rich repeat (NLR) proteins, which are characterized by a conserved N-terminal nucleotide-binding domain and a C-terminal leucine-rich repeat domain [1, 6, 7]. In the absence of pathogens, NLRs are typically maintained in an inactive state by interacting with other proteins *in planta*, whereas these interactions are disrupted in the presence of pathogens, prompting activation of the ETI response upon recognition of effectors [8]. However, the molecular mechanisms governing NLR homeostasis, particularly in crop species, remain poorly understood.

Increasing evidence indicates that the ubiquitin pathway is essential for ETI. For example, E3 ubiquitin ligase NbUBR7 interacts with the TNL N protein in tobacco and negatively regulates its stability, thereby mediating its resistance to *tobacco mosaic virus* [9]. By interacting with the NLR proteins SNC1 and RPS2, the F-box E3 ligase AtCPR1 mediates their degradation [10]. AtSGT1 interacts with the SKP1–CULLIN1–F-box (SCF) complex to facilitate the degradation of the *Arabidopsis* NLR and RPS5 proteins via the ubiquitination pathway [11–13]. Furthermore, SGT1 interacts with NSL1 (Necrotic Spotted Lesion 1), and the SGT1–NSL1 complex acts as a central regulatory module in NLR-mediated immune responses [14]. Many pathogen effectors have also been found to directly exhibit E3 ubiquitination activity or target host E3 ubiquitin ligases to suppress host immunity. For instance, AvrptoB from *Pseudomonas syringae* suppresses Pto-mediated cell death via autoubiquitination and E3 ligase activity [15]. P50, an effector of TMV, interferes with the interaction of NbUBR7, an N protein with E3 ligase, attenuates the degradation of N protein-enhanced defense signaling, and enhances resistance conferred by N proteins [9]. *Phytophthora sojae* Avr1d targets the soybean susceptibility factor GmPUB13 and inhibits the E3 ligase activity of GmPUB13 to stabilize GmPUB13 and facilitate *Phytophthora* infection [16].

Late blight, which is initiated by *Phytophthora infestans*, imposes the heaviest economic losses on global potato production [17]. *Phytophthora infestans* delivers numerous effectors into plant cells. These molecules are crucial for suppressing host defenses [18, 19]. For example, Avr3a stabilizes the host E3 ligase CMPG1 thereby inhibiting host cell death during the biotrophic phase of infection [20]. Avrblb2 targets the host papain-like cysteine protease C14 and specifically inhibits its secretion into the apoplast, thereby suppressing plant resistance [21]. Avr2 interacts with the positive regulator of potato BR signaling protein, BSL1 (BSU1-Like 1), to suppress plant immunity [22]. To date, several NLRs, such as R1, R2, R3a, R3b from *Solanum demissum*, Rpi-blb1, Rpi-blb2 from *Solanum bulbocastanum*, Rpi-vnt1 from *S. bulbocastanum* and Rpi-amr1, and Rpi-amr3 from *Solanum americanum* [6, 23, 24], have been shown to recognize these effectors for active ETI. *Rpi-amr3i* cloned from wild *S. americanum* confers broad-spectrum resistance to *P. infestans* [25]. Therefore, elucidating the regulatory mechanisms of NLRs and exploring new NLRs and their regulatory factors show great potential for combating late blight.

Although the function of *S* factors in plant immunity is more complex, they may provide an advantage in long-term resistance and adaptability by regulating the balance of the immune system and enhancing broad-spectrum resistance to multiple pathogens [26]*.* For example, *Rod1* (resistance of rice to diseases 1) encodes a novel calcium ion receptor that directly degrades reactive oxygen species, thereby suppressing immune responses in the absence of pathogen infection [27]. The susceptibility factor RTP1 is involved in regulating the endoplasmic reticulum stress signaling pathway during plant responses to *P. infestans* infection [28]. The protein tyrosine phosphatase StPTP1a suppresses salicylic acid (SA)-dependent immunity by interacting with and dephosphorylating StMPK4 and StMPK7 [29]. Two nonredundant blue light receptors, Stphot1 and Stphot2, independently negatively regulate plant resistance to *P. infestans* [30]. Therefore, the identification and utilization of *S* genes has significant potential for molecular breeding.

Previous studies have identified several potato E3 ubiquitin ligases function as regulators of innate immunity. For instance, two sugar transporters, StSWEET11 and StSWEET10c, facilitate *P. infestans* colonization by transporting sugars into the extracellular space. The ring-type E3 ubiquitin ligase StRFP1 interacts with StSWEET11 and StSWEET10c and facilitates their degradation through ubiquitination, thereby positively regulating plant resistance to *P. infestans* [31]. StFC-II (ferrochelatase 2) facilitates the infection of *P. infestans*, while the effector Pi22922 impedes the cytoplasmic degradation of StFC-II by the E3 ubiquitin ligase StCHIP, leading to its accumulation in chloroplasts and consequent suppression of host immunity [32]. However, the mechanisms by which the activity of potato E3 ubiquitin ligases is regulated to influence plant immunity have rarely been reported.

In this study, we identify StRWA2 (REDUCED WALL ACETYLATION 2) as a *P. infestans* susceptibility factor in potato that inhibits the accumulation of potato NLRs via the 26S proteasome, without significantly affecting cell wall acetylation as shown by acetic acid released assays, indicating that its role in susceptibility is independent of changes in cell wall acetylation. Furthermore, our results revealed that StRWA2 binds to the E3 ubiquitin ligase StSNIPER2 and relies on StSNIPER2 to inhibit NLR accumulation. Moreover, StRWA2 is able to increase the E3 ubiquitination activity of StSNIPER2, which also negatively regulates NLR-mediated HR and plant resistance against *P. infestans*. Finally, we obtained *StRWA2*-silenced potato plants via RNAi, and these plants exhibited increased resistance to *P. infestans*. Our findings provide new insights into NLR homeostasis regulation and identify novel *S* factors that show outstanding potential in combating *P. infestans*.

## Results

### RWA2s function as susceptibility factors in solanaceous plants

**Figure 1:**
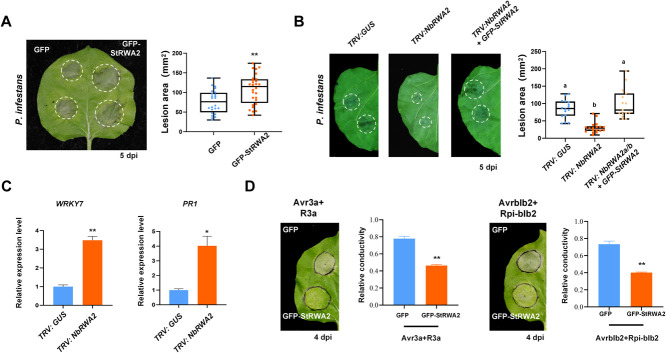
RWA2 negatively regulates plant resistance to *Phytophthora infestans*. (A) Infection phenotypes of *P. infestans* on *N. benthamiana* leaves expressing GFP-StRWA2. Images were captured at 5 dpi. Error bars indicate ±SDs, *n* ≥ 15 (^**^P < 0.01, Student’s *t* test). (B) Infection phenotypes of *P. infestans* on *NbRWA2*-silenced *N. benthamiana* leaves, in the presence or absence of GFP-StRWA2 co-expression. *TRV: GUS* served as a control. Images were captured at 5 dpi. Lesion area for *P. infestans* infection was shown by box plot. Standard deviations are displayed as error bars, *n* ≥ 15. Significant differences were displayed by different lowercase letters (*P* < 0.05, one-way ANOVA). (C) Relative expression levels of *WRKY7* and *PR1* in *NbRWA2*-silenced *N. benthamiana*. Total RNAs isolated from inoculated plants at 48 h. *EF1α* served as the reference for normalization. Error bars indicate the mean ± SEM of three independent biological replicates (^*^P < 0.05, ^**^P < 0.01, Student’s *t* test). (D) Transient expression of GFP-StRWA2 suppresses Avr3a/R3a- and Avrblb2/Rpi-blb2-triggered cell death. Images were captured at 4 dpi. Cell death was quantified by relative electrolytic leakage. Error bars indicate ±SDs, *n* = 3 (^**^P < 0.01, Student’s *t* test).

Previous research has established that RWA proteins are involved in cell wall acetylation and plant resistance to *Botrytis cinerea* [33, 34]. Here, we found that RWA2 is conserved in potato (*Solanum tuberosum*) and *Nicotiana benthamiana*, with its homologs designated as *StRWA2* (PGSC0003DMG400029061) and *NbRWA2* (NbD038958.1), respectively (Fig. S1). *Nicotiana benthamiana* is extensively used to elucidate the molecular mechanisms of solanaceous immunity against *P. infestans* [35–37]. To further investigate the function of *StRWA2,* we transiently expressed StRWA2 in *N. benthamiana*, and at 24 h after agroinfiltration, the leaves were inoculated with *P. infestans*. Larger lesions were observed on leaves expressing StRWA2, indicating that StRWA2 promoted *P. infestans* colonization (Fig. 1A). Furthermore, we also performed virus-induced gene silencing assay (VIGS) to silence *NbRWA2* in *N. benthamiana*, which led to a significant increase in resistance against *P. infestans* (Fig. 1B), and qRT-PCR analysis confirmed the effective silencing of *NbRWA2* (Fig. S2). We also found that the transcript levels of the defense-associated *pathogen-related* (*PR*) genes *PR1* and *WRKY7* were significantly upregulated in the *NbRWA2*-silenced plants (Fig. 1C)*.*

Additionally, complementation assays were conducted in *N. benthamiana* following silencing of endogenous *NbRWA2* (Fig. 1B). The results revealed that the overexpression of StRWA2 in *NbRWA2*-silenced *N. benthamiana* restored susceptibility, which promoted *P. infestans* infection. Collectively, these results indicate that RWA2s from *S. tuberosum* and *N. benthamiana* function as susceptibility factors that negatively regulate plant resistance to *P. infestans*. Moreover, we found that StRWA2 specifically suppressed the recognition of potato NLRs to *P. infestans* avirulence effector-mediated hypersensitive cell death, and the overexpression of StRWA2 impaired R3a/Avr3a- and Rpi-blb2/Avrblb2-triggered cell death but not PAMP INF1-triggered cell death in *N. benthamiana* (Fig. 1D and Fig. S3). Given that transient expression in potato is less efficient, we further employed a Potato virus X (PVX)-based VIGS system to knockdown *StRWA2* in cultivar ‘E10’, which carries the *R3a* gene. We found the Avr3a-triggered hypersensitive response (HR) occurred earlier and was more intense in *StRWA2*-silenced leaves, which was further confirmed by the significantly higher relative electrolyte leakage at 48 hpi (Fig. S4A–C). Furthermore, silencing *StRWA2* significantly enhanced potato resistance, as evidenced by markedly smaller lesion areas compared to the control (Fig. S4D). These results imply that StRWA2 negatively regulates plant resistance against *P. infestans* by suppressing NLR-mediated defenses.

To investigate the expression pattern of *StRWA2* during infection, we analyzed its expression patterns in two potato cultivars, E10 (containing *R3a*) and E3 (containing unidentified *NLR* genes), and used water treatment as a control to simulate the expression pattern under normal conditions. Results showed that in E10, *StRWA2* showed a rapid and continuous downregulation starting from 6 hpi, and in E3, *StRWA2* expression remained stable in the early stages and significantly decreases starting from 24 hpi, while remaining stable in mock-treated plants (Fig. S5). This consistent downregulation across different genetic backgrounds suggests that the suppression of the negative regulator *StRWA2* is a conserved and active defense response in potato during *P. infestans* challenge.

### *StRWA2*-silenced potato shows increased resistance to *P. infestans*

**Figure 2:**
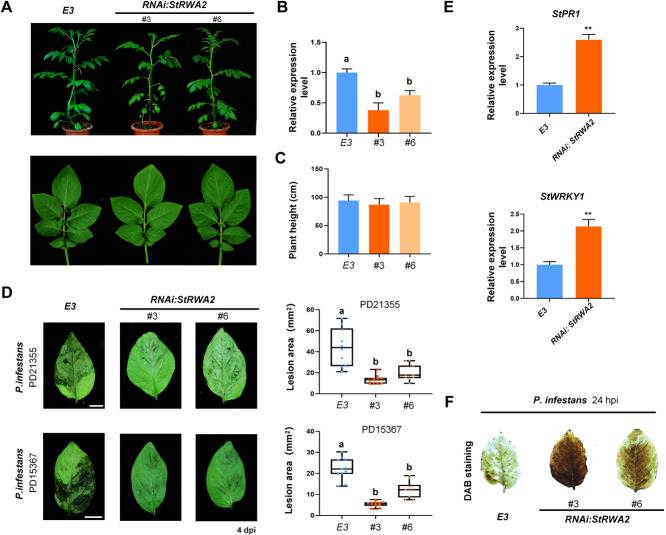
*StRWA2*-silenced potato plants show enhanced resistance to *P. infestans*. (A) Morphology of two *StRWA2*-RNAi-transgenic potato lines compared with control E3 at 6 weeks after planting. (B) qRT-PCR analysis of the *StRWA2* transcript level in RNAi plants compared with E3 plants. Error bars indicate the mean ± SEM of three independent biological replicates and significant differences were displayed by different lowercase letters (*P* < 0.05, one-way ANOVA). (C) Plant height statistics for E3 and *StRWA2*-silenced plants with error as standard deviation from three independent plants. (D) *Phytophthora infestans* infection phenotypes of *StRWA2*-silenced potato leaves. E3 served as a control. Standard deviations are displayed as error bars, *n* ≥ 10; significant differences were displayed by different lowercase letters (*P* < 0.05, one-way ANOVA). Scale bar = 1 cm. (E) Relative expression levels of *StPR1* and *StWRKY1* in *StRWA2*-silenced potato plants and control E3 plants. Total RNAs isolated from inoculated plants at 12 h. *StEF1α* served as the reference for normalization. Error bars indicate the mean ± SEM of three independent biological replicates (^**^P < 0.01, Student’s *t* test). (F) ROS accumulation in detached potato leaves after 24 h of *P. infestans* infection. Potato leaves stained with DAB.

Disrupting disease susceptibility *S* genes to interfere with pathogenicity mechanisms has led to substantial progress in molecular breeding techniques. Here, we obtained *StRWA2*-silenced potato via RNAi (RNA interference), which did not cause obvious developmental phenotype changes (Fig. 2A, B and Fig. S6). The qRT-PCR analysis confirmed effective silencing of *StRWA2* in RNAi transgenic plants (Fig. 2B). Three plants were measured for each line, and no significant difference in plant height was observed between *StRWA2*-silenced and E3 plants, suggesting that the absence of *StRWA2* in potato does not impair plant growth (Fig. 2C). To determine whether the silencing of *StRWA2* affects potato resistance to late blight, leaves from *StRWA2*-silenced plants were inoculated with *P. infestans* isolates PD21355 and PD15367, with E3 leaves serving as controls. At 5 dpi, the lesions on *StRWA2*-silenced potato plants were significantly smaller compared to those on E3 control (Fig. 2D). Furthermore, a qRT-PCR assay revealed the transcript levels of the *PR1* and *WRKY1* were significantly increased in the *StRWA2*-silenced plants (Fig. 2E). Moreover, DAB staining revealed that more ROS accumulated in *StRWA2*-silenced potato leaves than in the control E3 leaves (Fig. 2F). Taken together, these results demonstrate that silencing *StRWA2* in potato enhances plant resistance to *P. infestans*.

### StRWA2 destabilizes NLRs R3a and Rpi-blb2 through the 26S proteasome pathway

**Figure 3:**
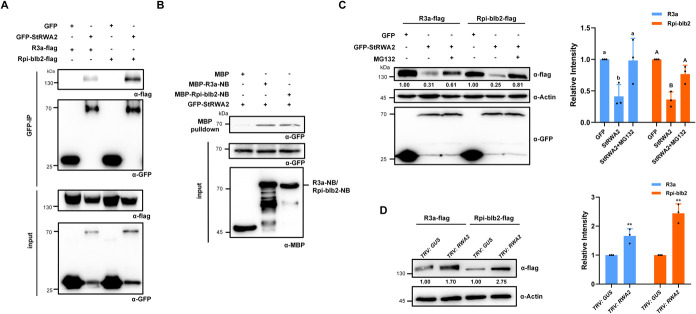
StRWA2 associates with R3a and Rpi-blb2 and facilitates their degradation via the 26S proteasome pathway. (A) Co-IP assay confirmed StRWA2 associates with R3a and Rpi-blb2. GFP or GFP-StRWA2 and R3a-flag or Rpi-blb2-flag were transiently expressed in *N. benthamiana*. Proteins were enriched using GFP-Trap beads. (B) *Semi*-*in vivo* pull-down assay confirmed StRWA2 associates with R3a and Rpi-blb2. GFP-StRWA2 was transiently expressed in *N. benthamiana*, MBP and MBP-R3a/Rpi-blb2-NB were expressed in Rosetta (DE3), and proteins were enriched using MBP-Trap beads. (C) StRWA2 facilitated R3a and Rpi-blb2 degradation in a 26S proteasome manner. GFP/GFP-StRWA2 and R3a-flag/blb2-flag were transiently expressed in *N. benthamiana*. Leaves were treated with 50 μM MG132 at 40 h. The bar chart shows the quantification of relative protein levels. Data represent the mean ± SD of three independent replicates. Significant differences were displayed by different lowercase letters (*P* < 0.05, one-way ANOVA). (D) Silencing of *NbRWA2* enhanced the accumulation of R3a and Rpi-blb2 in *N. benthamiana*. R3a-flag, and blb2-flag was transiently expressed in *TRV: GUS* and *TRV: NbRWA2* plants. α-Actin antibody was used to indicate the loading control. Relative gray values are calculated using ImageJ. Numbers indicate the relative intensity of R3a or Rpi-blb2 to plant actin normalized to GFP or *TRV: GUS* control. The bar chart shows the quantification of relative protein levels. Data represent the mean ± SD of three independent replicates (^**^P < 0.01, Student’s *t* test).

Since RWA proteins were reported to be associated with cell wall acetylation, we aimed to test whether *StRWA2/NbRWA2* affects cell wall acetylation and consequently plant immunity. To assess the cell wall acetylation, we measured the acetic acid released from cell wall preparations of both *N. benthamiana* and potato subjected to *RWA2* silencing, as well as from *N. benthamiana* leaves transiently expressing StRWA2. As shown in Fig. S7, neither silencing of *NbRWA2/StRWA2* nor transient expression of StRWA2 in *N. benthamiana* resulted in significant alterations in acetic acid released compared to the control. In contrast, the positive control (*AtRWA2-1*/*AtRWA2-2*) exhibited a significant reduction in acetic acid release, consistent with Manabe *et al.* [34]. These results indicate that silencing *StRWA2*/*NbRWA2* or transient expression of StRWA2 has no significant effect on cell wall acetylation. Given that StRWA2 has been shown to modulate the HR mediated by R3a/Rpi-blb2 and their corresponding Avr proteins, we carried out co-immunoprecipitation (Co-IP) assays to validate the associations of StRWA2 with R3a and Rpi-blb2. Our results revealed that R3a-flag and Rpi-blb2-flag were significantly enriched in GFP-StRWA2 precipitates but not in the GFP control (Fig. 3A and Fig. S8A). As the NB-ARC domain (Nucleotide-Binding adaptor shared by Apaf1, certain R genes, and CED4) is the conserved regulatory core responsible for the conformational switching of NLRs, we hypothesized that this domain might be the primary interface for RWA2 recruitment. To test this, we generated truncated variants of R3a and Rpi-blb2 encompassing their NB-ARC domains and performed *semi-in vivo* pull-down assay to test the interaction between StRWA2 with R3a and Rpi-blb2. The results showed that GFP-StRWA2 was significantly enriched in MBP-R3a-NB and MBP-Rpi-blb2-NB precipitates compared with the MBP control (Fig. 3B and Fig. S8B), confirming that StRWA2 associates with the NB-ARC domains of these NLRs. Since StRWA2 impairs the functions of the NLRs R3a and Rpi-blb2, we next investigated whether the abundances of R3a and Rpi-blb2 are affected by StRWA2. Therefore, R3a and Rpi-blb2 were co-expressed with GFP-StRWA2 or GFP in *N. benthamiana*, and their accumulation was detected by Western blot analysis. Results in Fig. 3C reveal that co-expression with GFP-StRWA2 resulted in markedly reduced accumulation of both R3a and Rpi-blb2 compared to co-expression with GFP (Fig. 3C and Fig. S8C). In addition, we confirmed a similar conclusion in *NbRWA2*-silenced *N. benthamiana*, in which R3a and Rpi-blb2 accumulated in *NbRWA2*-silenced plants (Fig. 3D and Fig. S8D)*.* Furthermore, the proteasomal inhibitor MG132-treated leaves exhibited enhanced accumulation of R3a and Rpi-blb2 (Fig. 3C and Fig. S8C). These findings demonstrate that StRWA2 promotes the degradation of the NLRs R3a and Rpi-blb2 *in planta* through the 26S proteasome pathway. Taken together, our results indicate that StRWA2 binds to NLRs to impair NLR-mediated cell death by destabilizing NLRs through the 26S proteasome pathway.

### StRWA2 binds to the susceptibility factor E3 ubiquitin ligase StSNIPER2

**Figure 4:**
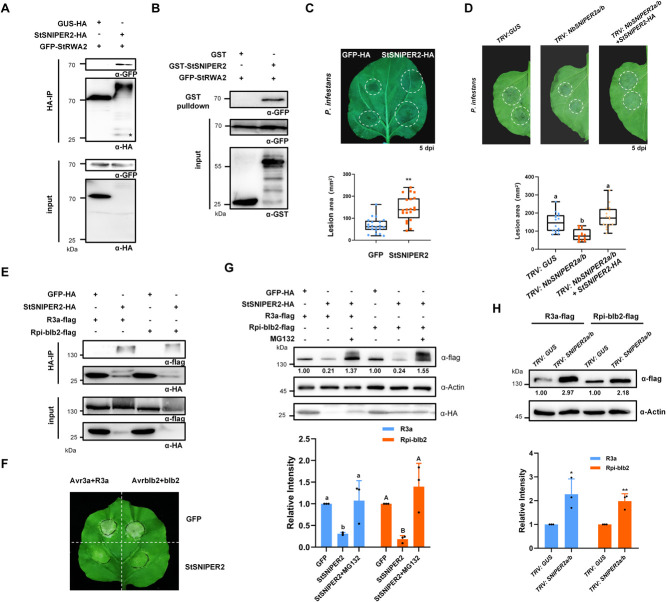
StRWA2 interacts with susceptibility factor StSNIPER2. (A) Co-immunoprecipitation (IP) assay confirmed StRWA2 associates with StSNIPER2. StSNIPER2-HA and GFP or GFP-StRWA2 were transiently expressed in *N. benthamiana*. Proteins were enriched using HA-Trap beads. (B) *Semi-in vivo* pull-down assay confirmed StRWA2 associates with StSNIPER2. GFP-StRWA2 was transiently expressed in *N. benthamiana*, GST and GST-StSNIPER2 were expressed in Rosetta (DE3), and proteins were enriched using GST-Trap beads. (C) Infection phenotypes of *P. infestans* on *N. benthamiana* leaves expressing StSNIPER2-HA. Images were captured at 5 dpi. GFP-HA infiltration was used as a control. Standard deviations are displayed as error bars, *n* ≥ 15 (^**^P < 0.01, Student’s *t* test). (D) Infection phenotypes of *P. infestans* on *NbSNIPER2*-silenced *N. benthamiana* leaves, in the presence or absence of StSNIPER2-HA co-expression. Images were captured at 5 dpi. Standard deviations are displayed as error bars, *n* ≥ 15. Significant differences were displayed by different lowercase letters (*P* < 0.05, one-way ANOVA). (E) Co-immunoprecipitation (IP) assay confirmed StSNIPER2 associates with R3a and Rpi-blb2. GFP-HA or StSNIPER2-HA and R3a-flag or Rpi-blb2-flag were transiently expressed in *N. benthamiana*. Proteins were enriched using HA-Trap beads. (F) Transient expression of StSNIPER2 in *N. benthamiana* suppresses Avr3a/R3a- and Avrblb2/Rpi-blb2-triggered cell death. Images were captured at 4 dpi. (G) StSNIPER2 facilitated R3a and Rpi-blb2 degradation in a 26S proteasome manner. GFP-HA or StSNIPER2-HA and R3a-flag or Rpi-blb2-flag were transiently expressed in *N. benthamiana* and treated with 50 μM MG132 at 40 h. The bar chart shows the quantification of relative protein levels. Data represent the mean ± SD of three independent replicates. Significant differences were displayed by different lowercase letters (*P* < 0.05, one-way ANOVA). (H) Silencing of *NbSNIPER2a/b* enhanced the accumulation of R3a and Rpi-blb2 in *N. benthamiana*. R3a-flag and Rpi-blb2-flag were transiently expressed in *TRV: GUS* and *TRV: NbSNIPER2a/b* plants. α-Actin antibody was used to indicate the loading control. Relative gray values are calculated using ImageJ. Numbers indicate the relative intensity of R3a or Rpi-blb2 to plant actin normalized to GFP or *TRV: GUS* control. The bar chart shows the quantification of relative protein levels. Data represent the mean ± SD of three independent replicates (^*^P < 0.05, ^**^P < 0.01, Student’s *t* test).

Given that StRWA2 promotes the degradation of NLRs (R3a and Rpi-blb2) via the proteasome pathway and specifically suppresses the HR mediated by Avr3a/R3a, Avr3b/R3b, Avr2/R2, and Avrblb2/Rpi-blb2 (Fig. S3), we hypothesized that StRWA2 may function by recruiting core components of the ubiquitin system. Since StRWA2 is not a typical component of the ubiquitin-proteasome system, we further proposed that this process depends on the involvement of an E3 ubiquitin ligase. We thus speculated that the potato homolog of AtSNIPER1/2 (an E3 ligase known to broadly modulate NLR stability [38]) would be a prime candidate for mediating StRWA2’s function. AtSNIPER1/2 has one homolog in potato (i.e. StSNIPER2) and two homologs in *N. benthamiana* (i.e. NbSNIPER2a/b) (Fig. S9). To validate the associations of StRWA2 with StSNIPER2, we carried out Co-IP assay. The result showed a significant enrichment of GFP-StRWA2 in StSNIPER2-HA immunoprecipitates compared to the GUS-HA control after transient co-expression in *N. benthamiana* (Fig. 4A). A reciprocal Co-IP assay further confirmed the interaction: StSNIPER2-HA was specifically enriched in GFP-StRWA2 immunoprecipitates compared to the GFP control (Fig. S10A). To further validate this interaction, we employed a *semi-in vivo* GST pull-down assay due to difficulties in expressing StRWA2 in *Escherichia coli*. Specifically, GFP-StRWA2 was expressed in *N. benthamiana* and total protein was incubated with GST or GST-StSNIPER2 purified from *E. coli*. As shown in Fig. 4B and Fig. S10B, GFP-StRWA2 was specifically enriched on GST-StSNIPER2-bound GST beads. Together, our results indicate that StRWA2 associates with the E3 ubiquitin ligase StSNIPER2.

To investigate the role of StSNIPER2 in late blight resistance, StSNIPER2-HA was transiently expressed in *N. benthamiana* with *P. infestans* inoculation performed 24 h post-agroinfiltration. At 5 dpi, leaves expressing StSNIPER2-HA exhibited larger lesions (Fig. 4C), demonstrating that StSNIPER2 serves as a negative regulator that promotes susceptibility to *P. infestans*. Furthermore, we silenced *NbSNIPER2a/b* in *N. benthamiana* using a conserved target region by VIGS (Fig. S11) and found that silencing of *NbSNIPER2a/b* increased the resistance to *P. infestans* (Fig. 4D)*.* Moreover, complementation assays further showed that expressing StSNIPER2 in *NbSNIPER2a/b*-silenced plants restored susceptibility to *P. infestans*, thereby functionally reconstituting the loss of *NbSNIPER2a/b* (Fig. 4D). To confirm these findings in potato, we employed a Potato virus X (PVX)-based VIGS system to silence *StSNIPER2* in *S. tuberosum*. Consistent with the results in *N. benthamiana*, *StSNIPER2*-silenced potato plants exhibited significantly smaller lesions upon *P. infestans* inoculation compared to the *PVX:GFP* control (Fig. S12A), with the silencing efficiency confirmed by qRT-PCR (Fig. S12B). Together, these findings demonstrate that both *S. tuberosum* and *N. benthamiana* SNIPER2 function as susceptibility factors that suppress plant immunity against *P. infestans*.

Since we previously observed that StRWA2 associates with R3a and Rpi-blb2 and suppresses R3a/Avr3a- and Rpi-blb2/Avrblb2-triggered cell death, we next verified and confirmed the interaction between StSNIPER2 and R3a and Rpi-blb2 via Co-IP (Fig. 4E and Fig. S10C) and found that the overexpression of StSNIPER2 suppressed R3a/Avr3a- and Rpi-blb2/Avrblb2-triggered cell death in *N. benthamiana* (Fig. 4F). Furthermore, *in vivo* protein stability assays demonstrated that StSNIPER2 and NbSNIPER2a/b facilitate the degradation of R3a and Rpi-blb2 through the 26S proteasome pathway (Fig. 4G, H, Figs S10D, E and S13). These results suggest that SNIPER2 negatively regulates plant resistance by destabilizing NLR proteins. Furthermore, to directly assess the impact of StRWA2 and StSNIPER2 on NLR accumulation in the native host, we transiently expressed R3a-flag and Rpi-blb2-flag in potato leaves where *StRWA2* or *StSNIPER2* had been silenced via PVX-VIGS. Although the protein expression efficiency in potato leaves is significantly lower than that in *N. benthamiana*, our result showed that the silencing of either StRWA2 or StSNIPER2 led to an enhanced accumulation of R3a and Rpi-blb2 compared to the control (Fig. S12C). This result further reinforces our biochemical model in the potato context.

### StRWA2 enhances the E3 ligase activity of StSNIPER2

**Figure 5:**
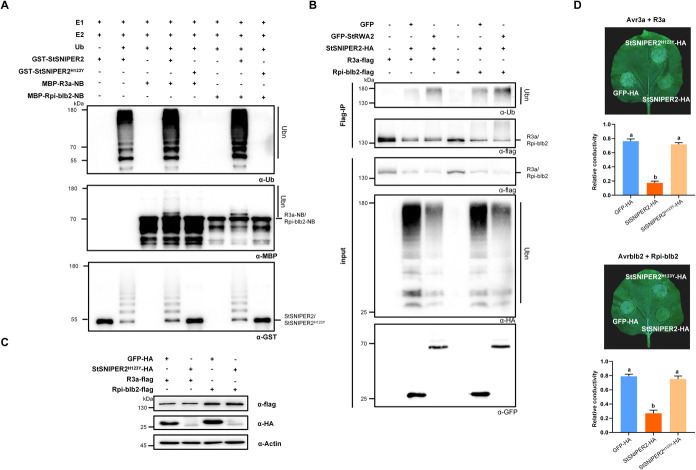
StSNIPER2 exhibits StRWA2-enhanced E3 ligase activity and suppresses R3a/Avr3a- and Rpi-blb2/Avrblb2-triggered cell death. (A) *In vitro* ubiquitination assays demonstrated that R3a and Rpi-blb2 are ubiquitinated by StSNIPER2. (B) StRWA2 enhances StSNIPER2-mediated ubiquitination of R3a and Rpi-blb2 *in vivo*. Flag-tagged R3a or Rpi-blb2 was co-expressed with StSNIPER2HA and GFP-StRWA2 in *N. benthamiana*. Leaves were treated with 50 μM MG132 at 2 h before harvest. Following immunoprecipitation with Flag-Trap beads, ubiquitination levels were detected using an anti-Ub antibody. (C) StSNIPER2^H123Y^ does not affect the stability of R3a or Rpi-blb2. GFP-HA or StSNIPER2^H123Y^-HA and R3a-flag or Rpi-blb2-flag were transiently expressed in *N. benthamiana* and harvested at 48 h. α-Actin antibody was used to indicate the loading control. (D) StSNIPER2 suppresses R3a/Avr3a- and Rpi-blb2/Avrblb2-triggered cell death in a ubiquitin activity-dependent manner. Cell death was quantified by relative electrolytic leakage. Standard deviations are displayed as error bars, *n* = 3; significant differences were displayed by different lowercase letters (*P* < 0.05, one-way ANOVA).

To assess the ubiquitin ligase activity of StSNIPER2, an *in vitro* ubiquitination assay was conducted. Ubiquitin immunoblotting revealed a characteristic smearing pattern, indicating the formation of polyubiquitin chains of varying molecular weights. In contrast, reactions containing only E1, E2, and GST or reactions that did not contain Ub showed no smearing (Fig. 5A and Fig. S14A), indicating that StSNIPER2 is an active ubiquitin ligase. The RING domain residue histidine (H) 129 of AtSNIPER2 has been identified as critical for its ubiquitin ligase function [38]. This residue is conserved in StSNIPER2, and the corresponding residue is H123. We generated the StSNIPER2^H123Y^ mutant and found that it had lost ubiquitin ligase activity, based on the absence of polyubiquitin smearing (Fig. 5A and Fig. S14A). To determine whether StRWA2 influences the ligase activity of StSNIPER2, GFP-StRWA2 was added to the reaction mixture. This addition resulted in enhanced polyubiquitin smearing compared to control conditions (Fig. S14A). Furthermore, we performed *in vivo* ubiquitination experiments. Consistent with our *in vitro* findings, leaves co-expressing StSNIPER2 and StRWA2 exhibited more extensive polyubiquitin smearing compared to those expressing StSNIPER2 and GFP (Fig. S14B). Moreover, StSNIPER2 was transiently expressed in *NbRWA2*-silenced plants, and a marked reduction in polyubiquitin smearing was observed relative to the control (Fig. S14C). Overall, our results indicate that RWA2s promote the auto-ubiquitination of StSNIPER2 both *in vitro* and *in vivo*.

We further investigated whether StSNIPER2 targets the NLR proteins R3a and Rpi-blb2 for ubiquitination and conducted *in vitro* ubiquitination assays using the NB-ARC domains of R3a and Rpi-blb2 as substrates. Immunoblotting revealed a characteristic high-molecular-weight smearing pattern for both substrates, indicating that StSNIPER2 directly polyubiquitinates the NB-ARC domains of R3a and Rpi-blb2 (Fig. 5A and Fig. S15A). In contrast, no such smearing was observed in control reactions lacking StSNIPER2, confirming its substrate-specific ligase activity. Correspondingly, *in vivo* ubiquitination assays revealed that R3a and Rpi-blb2 exhibited more extensive polyubiquitin smearing when co-expressed with StSNIPER2 compared to the GUS-HA control (Fig. S16). To further determine whether StRWA2 influences this process, GFP-StRWA2 was co-expressed and resulted in significantly enhanced polyubiquitin smearing of both R3a and Rpi-blb2 compared to the StSNIPER2-only control (Fig. 5B and Fig. S15B). Collectively, these results demonstrate that StSNIPER2 acts as an E3 ligase to ubiquitinate the NB-ARC domains of R3a and Rpi-blb2, and StRWA2 functions as a crucial co-regulator that promotes this ubiquitination process.

### StSNIPER2 interacts with and destabilizes R3a and Rpi-blb2 depending on its E3 ligase activity

Our previous result revealed that StSNIPER2 promotes the degradation of R3a and Rpi-blb2; therefore, to determine whether this E3 ligase mediates the degradation of R3a and Rpi-blb2, we tested the protein levels of R3a and Rpi-blb2 via agroinfiltration in *N. benthamiana* when co-expressed with StSNIPER2^H123Y^ *in vivo*. As shown in Fig. 5D and Fig. S17, both R3a and Rpi-blb2 accumulation did not show significant changes in the presence of StSNIPER2^H123Y^. Furthermore, we found that the overexpression of StSNIPER2^H123Y^ failed to suppress R3a/Avr3a- and Rpi-blb2/Avrblb2-mediated cell death, and a relative electrolytic leakage assay confirmed this phenotype (Fig. 5E). These findings suggest that the ability of StSNIPER2 to degrade R3a and Rpi-blb2 and suppress NLR-mediated cell death depends on its ligase activity.

### SNIPER2 is required for the susceptibility role of StRWA2

**Figure 6:**
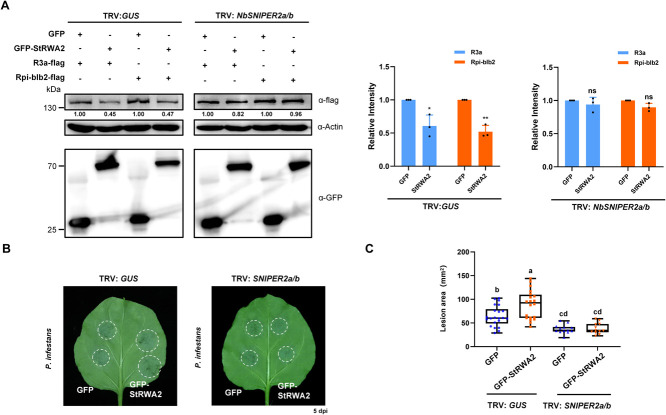
SNIPER2 is required for the susceptible function of StRWA2. (A) StSNIPER2 is essential for the StRWA2-mediated destabilization of R3a and Rpi-blb2. GFP or GFP-StRWA2 and R3a-flag or blb2-flag were transiently expressed in *TRV: GUS* and *TRV: NbSNIPER2a/b* plants. Proteins were detected with anti-flag, anti-GFP antibodies, and anti-actin antibody was used to indicate the loading control. Relative gray values are calculated using ImageJ. Numbers indicate the relative intensity of R3a or Rpi-blb2 to plant actin normalized to GFP control. The bar chart shows the quantification of relative protein levels. Data represent the mean ± SD of three independent replicates (^*^P < 0.05, ^**^P < 0.01, Student’s *t* test). (B) Representative images showing the lesions in *TRV: GUS* and *TRV: NbSNIPER2a/b* leaves that developed on GFP and GFP-StRWA2 agro-infiltrated. Images were captured at 5 dpi. (C) Lesion area for *P. infestans* infection was shown by boxplot, and standard deviations are displayed as error bars. *n* ≥ 12. Significant differences were displayed by different lowercase letters (*P* < 0.05, one-way ANOVA).

We previously observed that both StRWA2 and StSNIPER2 are able to destabilize NLRs R3a and Rpi-blb2 and negatively regulate plant resistance. To investigate whether this function of StRWA2 relies on the presence of SNIPER2, we silenced *NbSNIPER2a/b* in *N. benthamiana* via TRV-mediated VIGS and then co-expressed StRWA2 with R3a and Rpi-blb2 in *NbSNIPER2a/b-*silenced plants. The results revealed that the effect of StRWA2 on R3a and Rpi-blb2 degradation was significantly attenuated in *NbSNIPER2a/b*-silenced *N. benthamiana* compared with the control (Fig. 6A and Fig. S18), suggesting that StRWA2 binds to and utilizes the E3 ubiquitin ligase SNIPER2 to destabilize NLRs. Furthermore, we tested whether StRWA2-mediated plant susceptibility was altered in the absence of *NbSNIPER2a/b*. GFP and GFP-StRWA2 were transiently expressed in *TRV*: *GUS* and *TRV: NbSNIPER2a/b* plants. Silencing of *NbSNIPER2a/b* decreased plant susceptibility to *P. infestans* mediated by StRWA2 relative to the control (Fig. 6B and C). These results indicate that SNIPER2 is required for the susceptibility role of StRWA2*.*

## Discussion

Resistance to late blight is achieved via potato breeding by introducing *R* genes that are effective against different virulent isolates; however, *P. infestans* can rapidly escape *R* gene-mediated resistance [39–41]. Disruption of *S* genes confers more durable and broad-spectrum resistance compared to the introduction of *R* genes. Several *S* factors have been identified in plants, notably in the model plant *Arabidopsis* (i.e. RIN4, Receptor-like kinase IOS1, bHLH25/27, etc.) [42–44]. Although *S* factors provide promising targets for breeding due to their disruption leading to reduced susceptibility, far fewer have been characterized in potato compared to model plants like *Arabidopsis* or crops like rice. Nonetheless, several potato *S* factors have been identified; for example, the putative K-homology (KH) RNA-binding protein, StKRBP1, depends on the nucleotide-binding ability of its KH domain to enhance *P. infestans* infection [45]. StNRL1 (NPH 3/RPT 2-LIKE 1) inhibits the cell death induced by INF1 and enhances *P. infestans* colonization [46, 47]. The L-type lectin receptor-like kinase StLecRK-IV.1 interacts with StTET8, a positive immune modulator, promoting its degradation [48]. In this study, we identify StRWA2 as a novel susceptibility factor in potato that negatively regulates NLR-mediated resistance to *P*. *infestans* (Figs 1 and 2).

Although previous studies in *Arabidopsis* have shown that RWA2 is a cell wall-associated protein that plays important roles in plant immunity, our findings reveal a novel, immunity-related function independent of this role. The *AtRWA2* mutant released about 20% less acetic acid upon alkali treatment than the wild type, indicating reduced cell wall acetylation, and exhibited enhanced resistance to *B. cinerea* [34]. However, our results show that neither silencing *StRWA2* in potato nor suppressing or overexpressing its ortholog in *N. benthamiana* led to significant changes in acetic acid released, and *AtRWA2-1*/*AtRWA2-2* was used as a positive control (Fig. S7). We speculated that the absence of an observable change in acetylation may result from functional redundancy among RWA homologs or the limited contribution of *StRWA2* to overall cell wall acetylation. Instead, StRWA2 promotes susceptibility to *P. infestans* by binding an E3 ubiquitin ligase to facilitate NLR destabilization and degradation (Figs 3 and 4). Thus, we demonstrate that StRWA2 plays a distinct and noncanonical role unrelated to cell wall acetylation during the *P. infestans* infection.

The homeostasis of NLR proteins is precisely monitored by multiple conserved mechanisms, which collectively ensure their timely activation and proper suppression in plant immunity, thereby maintaining the effectiveness and safety of the immune response. While NLRs are indispensable for pathogen recognition and defense activation, their uncontrolled accumulation triggers deleterious autoimmunity and growth suppression—a phenomenon well-documented in *Arabidopsis* [49, 50]. This duality necessitates robust regulatory mechanisms to balance immune activation and physiological fitness. Among the key players in NLR homeostasis are E3 ubiquitin ligases, which orchestrate targeted degradation of NLRs through the 26S proteasome pathway. For instance, the antagonistic U-box ligases PUB5/PUB44 dynamically regulate SUMM2-mediated autoimmunity [51], while RING-type ligases like AtMUSE16 directly target RPS2 for degradation to prevent immune overactivation [52]. Previous work has shown that *Arabidopsis* SNIPER1/2 facilitates the degradation of multiple NLRs, such as SNC1, SUMM2, RPS4, RPP4, RPS2, and RPM1 [38]. The homolog of AtSNIPER1/2 in *N. benthamiana*, NbSNIPER2a/b, targets and degrades the positive immune regulator BIR2 (BAK1-INTERACTING RLK 2), thereby negatively regulating PTI [53]. Our results indicate that NbSNIPER2a/b also destabilizes the NLRs, such as R3a and Rpi-blb2, revealing the multiple functions of SNIPER2 in the plant immune system (Fig. 4H and Fig. S13). Thus, our study not only extends the paradigm of NLR regulation by SNIPER2 to solanaceous crops but also identifies StRWA2 as a novel regulator in this conserved mechanism.

Most E3 ubiquitin ligases, particularly RING-type E3 ubiquitin ligases, possess autoubiquitination activity [54, 55]. In this study, both *in vitro* and *in vivo* experiments demonstrated that StSNIPER2 auto-ubiquitinates and regulates its own stability (Fig. S14A and B). When the histidine at position 123 was mutated to a tyrosine, the degradation of R3a and Rpi-blb2 by StSNIPER2 and its auto-ubiquitination were significantly attenuated (Fig. 5C). Furthermore, we observed that StRWA2 significantly enhances the ubiquitination activity of StSNIPER2, as evidenced by increased polyubiquitination levels in both plant-based and reconstituted experimental systems in the presence of StRWA2. Interestingly, although StRWA2-promoted auto-ubiquitination of StSNIPER2 reduces its own stability (Fig. 5B and Fig. S14A, B), within a certain range of degradation, StSNIPER2 remains capable of effectively facilitating the degradation of NLRs. Notably, the precise mechanism by which StRWA2 activates StSNIPER2 remains unclear. It is plausible that StRWA2 may recruit additional co-factors or interact with other proteins that collectively modulate SNIPER2’s E3 ligase activity. Further investigation is needed to determine whether StRWA2 acts solely through direct interaction with StSNIPER2 or requires auxiliary components for full functionality.

Damage to *S* factors may also result in impaired growth or cause adaptive penalties similar to those associated with autoimmunity. For example, silencing the *S* genes *StDMR1* and *StDND1* caused a certain degree of growth phenotype deterioration in plants [56]. Therefore, the balance between plant immunity and growth should also be considered in resistance breeding [26, 57]. *MLO* showed durable and broad-spectrum resistance to powdery mildew. One mlo-edited line exhibits an unintended genomic deletion that upregulates TaTMT3B, a vacuolar monosaccharide transporter gene, resulting in the recovery of growth and yield [58, 59]. In our study, *StRWA2-*silenced potato lines generated via RNAi did not exhibit impaired growth and showed enhanced resistance to *P. infestans* with different isolates, revealing potential applications in potato resistance breeding (Fig. 2). One plausible explanation for this lack of growth penalty is the unique self-regulatory mechanism of the StRWA2-StSNIPER2 module. Our data indicate that StRWA2 does not target NLR stability directly, but rather modulates the enzymatic activity of the E3 ligase StSNIPER2. Crucially, StSNIPER2 undergoes auto-ubiquitination to regulate its own homeostasis (Fig. 5B and Fig. S14A, B). When StRWA2 enhances StSNIPER2’s ligase activity, it concurrently promotes StSNIPER2’s own degradation, thereby preventing an excessive accumulation of the E3 ligase that might otherwise lead to a catastrophic depletion of NLRs or other essential immune components. This internal feedback loop likely buffers the plant against the deleterious effects of immune overactivation, maintaining a fine-tuned balance between defense and development. This strategy could lead to durable resistance, bypassing the arms race between NLRs and rapidly evolving effectors.

**Figure 7:**
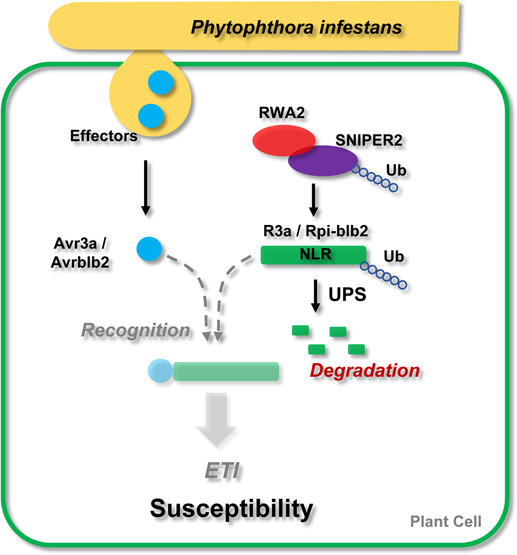
A proposed model showing that StRWA2 recruits the E3 ubiquitin ligase StSNIPER2 to degrade NLRs, thereby suppressing effector-triggered immunity mediated by Avr–NLR recognition and promoting plant susceptibility.

Our findings support a model in which StRWA2 serves as a regulator of ETI and disease resistance (Fig. 7). In this model, NLR must be precisely regulated to prevent autoimmunity and fitness costs. StRWA2 recruits the E3 ubiquitin ligase StSNIPER2 to enhance its enzymatic activity, thereby forming a regulatory module for NLR degradation. Interestingly, we observed that *StRWA2* expression is significantly suppressed following *P. infestans* infection in both E10 and E3 potato cultivars. The rapid and continuous downregulation of this negative regulator upon pathogen challenge suggests an active defense strategy in potato, potentially facilitating the rapid accumulation of NLR proteins (such as R3a) to efficiently activate ETI. Furthermore, the susceptibility function of StRWA2 depends on SNIPER2. This regulatory mechanism helps to maintain NLR homeostasis, allowing plants to balance defense responses and normal growth. When *RWA2*/*SNIPER2* is silenced, SNIPER2-mediated NLR degradation is compromised, leading to the accumulation of NLRs and enhanced resistance to *P. infestans*. This model not only provides a novel perspective for understanding the balance of plant immunity but also offers a theoretical foundation for achieving broad-spectrum disease resistance breeding through the editing of susceptibility genes.

In this study, R3a and Rpi-blb2 were utilized as representative models because they are well-characterized NLRs with established cognate effectors. While *N. benthamiana* and the potato cultivars used may not harbor these specific *R* genes, they contain numerous other endogenous NLR genes that remain to be identified and characterized. Our results in the potato cultivar E10, which naturally carries the R3a gene, provide direct evidence for this regulatory mechanism in a native host background. By silencing *StRWA2* in E10 via PVX-VIGS, we observed a significantly accelerated HR upon Avr3a infiltration and enhanced resistance to *P. infestans*. This confirms that StRWA2 indeed functions to destabilize endogenous NLRs to modulate immunity. Crucially, our experimental data show that StRWA2 also strongly suppresses the hypersensitive response (HR) mediated by other NLRs, such as R2 and R3b. This demonstrates that the RWA2–SNIPER2 complex exerts a broad regulatory effect across the NLR family, rather than being limited to specific receptors. This broad activity is further supported by our biochemical evidence, which shows that StSNIPER2 directly ubiquitinates the NB-ARC domain-a signaling module highly conserved across almost all plant NLRs. Therefore, it is highly probable that this module regulates a wide range of unidentified endogenous NLRs present in the host plants. Notably, while the RWA2–SNIPER2–NLR axis represents a major mechanism underlying potato susceptibility, the complex regulatory roles of *S*-genes suggests that additional hitherto unknown resistance mechanisms might be triggered upon StRWA2 disruption. These may include the stabilization of non-NLR immune regulators or the activation of complementary defense signaling pathways. Future studies may focus on elucidating the precise molecular mechanism by which StRWA2 regulates StSNIPER2 and exploring these potential unknown mechanisms to fully harness the power of *RWA2*-mediated resistance in crop improvement.

## Materials and methods

### Plasmid construction

The vectors were constructed utilizing ClonExpress II One Step Cloning Kit (Vazyme, Nanjing, China). For transient expression, *StRWA2* was constructed with an N-terminal GFP tag in the pCAMBIA1300 vector. *StSNIPER2* and *StSNIPER2^H123Y^* were constructed in the pCAMBIA1300 vector with a C-terminal HA tag. *R3a* and *Rpi-blb2* were constructed in the pCAMBIA1300 vector with a C-terminal flag tag. For prokaryotic expression, *StSNIPER2* or *StSNIPER2^H123Y^* were constructed with an N-terminal GST tag in the pGEX4T-3 vector. *R3a* and *Rpi-blb2* were constructed in the pMAL-c5x vector with a C-terminal MBP tag. For virus-induced gene silencing, *NbRWA2* and *NbSNIPER2a/b* were cloned into pTRV2. The primers are listed in Table S1.

### Plant materials and growth conditions

*Nicotiana benthamiana* and potato (*S. tuberosum* L) plants were grown at 23°C with a 16-h light/8-h dark photoperiod. The growth substrate was prepared by mixing loam soil and vermiculite in equal volumes. Seedlings of the RNA interference (RNAi) potato cultivar ‘E-Potato-3’ (‘E3’) were germinated in tissue culture vessels on standard MS medium (3% sucrose, 0.7% agar). Subsequently, plants were moved to a growth chamber set at 23°C with a 16-h light/8-h dark cycle. The E3 potato plants were then transplanted into a glasshouse and grown under natural environmental light conditions. After 6 weeks of growth in the glasshouse, these plants were utilized for *P. infestans* infection assays. Two *AtRWA2* mutants, *AtRWA2-1* and *AtRWA2-2* (SALK_003787 and SALK_078630), were acquired from the SALK mutant library.

### Virus-induced gene silencing assay

The TRV (Tobacco rattle virus) system was employed for gene silencing in *N. benthamiana.* Equal volumes of *Agrobacterium tumefaciens* containing pTRV2-*RWA2*, pTRV2-*SNIPER2*, and pTRV2-*PDS* with pTRV1 were mixed. And the four-leaf stage *N. benthamiana* were infiltrated. The *PDS* genes that produced the albino phenotype after 3–4 weeks were used as visual markers, and the VIGS efficiency was detected by qRT-PCR. The PVX (*Potato virus X*) system was employed for gene silencing in potato plants [60]*.* The *StSNIPER2* fragment was cloned into the pGR106 vector (a PVX-based vector). *Agrobacterium tumefaciens* carrying either pGR106-*StSNIPER2* or pGR106-*GFP* (negative control) were prepared. The final concentration of the *Agrobacterium* was adjusted to an OD_600_ of 0.5 in infiltration buffer and incubated at room temperature for 3 h before inoculation. Four-week-old potato plants were used for infiltration. Approximately 3–4 weeks after inoculation, newly developed leaves were collected for qRT-PCR analysis to determine the silencing efficiency of *StSNIPER2*.

### *Phytophthora infestans* infection and inoculation assay

*Phytophthora infestans* strains were cultured on rye agar plates at 18°C in darkness for 12 days. To collect sporangia, 5 mL of ice-cold sterile water was added to each plate, and the surface was gently scraped with a cell spreader. The resulting suspension was collected and pipetted into a 50-ml centrifuge tube. After centrifugation (3,000 rpm, 5 min, 10°C), the supernatant was carefully aspirated and discarded. The pellet was then gently resuspended in the remaining ~200 μl of liquid, and 2 ml of sterile distilled water was added to the tube. For inoculation, 10-μL droplets containing approximately 350 sporangia (for *N. benthamiana*) or 200 sporangia (for potato) were inoculated onto the leaves. Inoculated plants were maintained at 18°C in darkness for 24 h to facilitate infection, then transferred to a growth chamber with a 16-h light/8-h dark photoperiod. Lesion areas were quantified using ImageJ at  3-5 dpi.

### RNA extraction and qRT-PCR analysis

Total RNA was extracted with Trizol reagent (Invitrogen, Carlsbad, CA, USA). cDNA was reverse transcribed from 1 μg of RNA with the Genestar RT Master Mix (Beijing, China). qRT-PCR was performed using SYBR Green Master Mix (Genestar). Relative expression levels were quantified using the 2^ΔΔ^Ct method with *NbEF1α* or *StEF1α* serving as reference genes for normalization.

### Inhibition of HR *in planta*

*GFP-StRWA2* or *GFP* was transiently expressed in *N. benthamiana*. After 24 h, pGR106-Avr3a/R3a-flag or pGR106-Avrblb2/Rpi-blb2-flag was co-infiltrated into the same region of the leaves at a final OD_600_ of 0.3. Cell death was monitored between 2 and 4 days postinfiltration. Each replicate consisted of five plants, with three leaves per plant analyzed. HR was quantified by assessing leaf sector collapse, where lesions encompassing ≥50% of the infiltrated area were scored as HR positive and <50% as negative. In the case of successful HR inhibition, these HR symptoms should be significantly reduced or absent. The leaf phenotypes were photographed at regular intervals, such as 1, 2, 3, and 5 days postinfiltration.

### Electrolyte leakage measurement and DAB staining

After floating on distilled water for 3 h, four leaf discs were boiled for 20 min and subsequently cooled to 25°C. Relative electrolyte leakage was quantified by measuring conductivity ratio between unboiled and boiled leaf discs with a conductivity meter. DAB staining was performed by incubating leaf tissues in a 1 mg/ml solution under dark conditions for 8 h and then decolorized in 95% ethanol.

### Western blot assay

Plant tissue was frozen in liquid nitrogen and subsequently pulverized. Add 70 μL protein extraction buffer (1 M Tris–HCl, 3 M NaCl, 0.5 M EDTA, 1 mM DTT, 1 mM cocktail, 0.1% Triton X-100, pH 7.5) per 0.1 g of plant tissue. The proteins were incubated in 95°C for 5 min in SDS-loading buffer (StRWA2 was incubated in 65°C for 5 min). Anti-GFP (TransGen Biotech, Beijing, China), anti-HA (TransGen Biotech, Beijing, China), anti-flag (Sigma-Aldrich, St. Louis, MO, USA), and goat anti-mouse antibody was used according to descriptions in the manual. Anti-actin antibody (Kangti Life Sciences, Beijing, China) was used to verify equal loading.

### Cell wall preparation and quantitative analysis of acetyl esters

To determine cell wall acetylation by measuring acetic acid release, we prepared the alcohol-insoluble residue (AIR) as previously described [61]. After saponification of 10 mg AIR in 0.09 M sodium hydroxide solution overnight, 1 M hydrochloric acid solution was added to terminate the reaction. The supernatant was collected and analyzed for acetic acid release using Acetic Acid Assay Kit (Megazyme, Wicklow, Ireland).

### Protein stability *in planta*

To determine whether StRWA2 or StSNIPER2 affects the stability of R3a or Rpi-blb2, R3a-flag or Rpi-blb2-flag was co-expressed with either StSNIPER2-HA or GFP-HA (control), or with GFP-StRWA2 or GFP (control) for 40 h. The experimental groups were treated with 50 μM MG132 (MedChemExpress, Monmouth Junction, NJ, USA) 4 h before harvest. A control group was treated with dimethyl sulfoxide (DMSO) diluted to 5% with ddH_2_O under the same conditions. To assess the impact of silencing *NbRWA2*/*NbSNIPER2* on the stability of R3a/Rpi-blb2, R3a-flag or Rpi-blb2-flag was transiently expressed in *TRV: RWA2* or TRV: *SNIPER2s* and TRV: *GUS N. benthamiana* for 40 h, followed by treatment with 50 μM CHX (MedChemExpress, Monmouth Junction, NJ, USA) 2 h before harvest. Protein stability was detected by Western blot assay. Relative gray values were quantified with ImageJ. The relative intensity of R3a or Rpi-blb2 to plant actin was normalized to the GFP control.

### Co-immunoprecipitation and *semi-in vivo* pull-down assay

For co-immunoprecipitation assay, proteins were transiently expressed in *N. benthamiana* for 48 h and enriched with 15 μL GFP or HA-Trap beads (Kangti Life Sciences, Beijing, China) for 2 h and washed at least three times. The interaction was detected by Western blot after incubating at 65°C for 10 min. For *semi-in vivo* pull down assay, *GFP-StRWA2* was expressed in *N. benthamiana*, then 1 mL extraction buffer was added for cell lysis. Approximately 2 μg GST/GST-StSNIPER2 or MBP/MBP-R3a/Rpi-blb2 was enriched with 20 μL GST/MBP-Trap beads (Cytiva, Marlborough, MA, USA) for 2 h. Subsequently, GFP-StRWA2 lysate was added to GST/MBP-Trap beads prebound with GST/GST-StSNIPER2 or MBP/MBP-R3a/Rpi-blb2 and incubated for 1 h. The beads were washed at least five times with PBS. The interaction was detected by Western blot after incubating at 65°C for 5 min.

### *In vitro* and *in vivo* ubiquitination assay

For *in vitro* ubiquitination assay, ubiquitin, ubiquitin-activating enzyme (E1), ubiquitin-conjugating enzyme (E2), GST, GST-StSNIPER2, and GST-StSNIPER2^H123Y^ were purified from *E. coli*. GFP and GFP-StRWA2 were transiently expressed in *N. benthamiana* and enriched with 15-μl GFP-Trap beads. Each reaction contained 2 μg ubiquitin, 100 ng E1, 1 μg E2, 1 μg GST-StSNIPER2 or GST-StSNIPER2^H123Y^, and 1 μg MBP-R3a-NB or MBP-Rpi-blb2-NB in ubiquitination buffer (50 mM Tris–HCl, 5 mM MgCl_2_, 2 mM DTT, 2 mM ATP) for 1.5 h. Add SDS loading buffer to terminate the reaction. Incubate at 95°C for 5 min and detect ubiquitination using an anti-Ubiquitin (Ub) antibody (Abcam, Cambridge, UK) by Western blot. To perform *in vivo* ubiquitination assay, proteins were transiently expressed in *N. benthamiana*. To facilitate the accumulation of ubiquitinated intermediates, leaves were treated with 50 μM MG132 for 2–4 h before harvest. Total protein was extracted and was enriched using flag-Trap beads. Ubiquitination signals were detected with anti-Ub antibody.

### Potato transformation

The 205 bp *StRWA2* fragment from potato cDNA sequences was cloned into the pHellsgate8 vector. The potato cultivar E3 was transformed following the method described previously [62].

## Supplementary Material

Web_Material_uhag072

## Data Availability

All data are available in the manuscript or the supplementary material.
