# Supplementary material for: Potato StRWA2 recruits the E3 ligase StSNIPER2 to destabilize NLRs and promote Phytophthora infestans infection
Source: Hortic Res. 2026 Mar 3;13(7):uhag072. doi: 10.1093/hr/uhag072 (PMC13267645; doi:10.1093/hr/uhag072)
Supplement: Web_Material_uhag072 [file Web_Material_uhag072.docx]

**Supplemental information**

**Potato RWA2 recruits the E3 ligase SNIPER2 to destabilize NLRs and promote *Phytophthora infestans* infection**

Zhengyu Chen^1^, Ziwei He^1^, Juan Du^1^, Yuhe Li^1^, Siqi Niu^2^, Aifang Ma^3^, Qian Chen^1^, Hailong Guo^1^, Jun Fan^1^, Maozhi Ren^4^, Guangyuan Xu^1^, Daolong Dou^1,2^, Jinguang Yang^5^, Maofeng Jing^2*^, Xiaodan Wang^1*^

1.State Key Laboratory of Agricultural and Forestry Biosecurity, MOA Key Lab of Pest Monitoring and Green Management, College of Plant Protection, China Agricultural University, Beijing 100193, China

2.State Key Laboratory of Agricultural and Forestry Biosecurity, College of Plant Protection, Nanjing Agricultural University, Nanjing, 210095, China

3.College of Biological Sciences, China Agricultural University, Beijing, 100193, China

4.Institute of Urban Agriculture, Chinese Academy of Agricultural Sciences, Chengdu National Agricultural Science and Technology Center, Chengdu, 610000, China

5. Key Laboratory of Tobacco Pest Monitoring Controlling & Integrated Management, Tobacco Research Institute of Chinese Academy of Agricultural Sciences, Qingdao 266101, China

*Correspondence email: Xiaodan Wang, xdwang@cau.edu.cn; Maofeng Jing, jingmf@njau.edu.cn


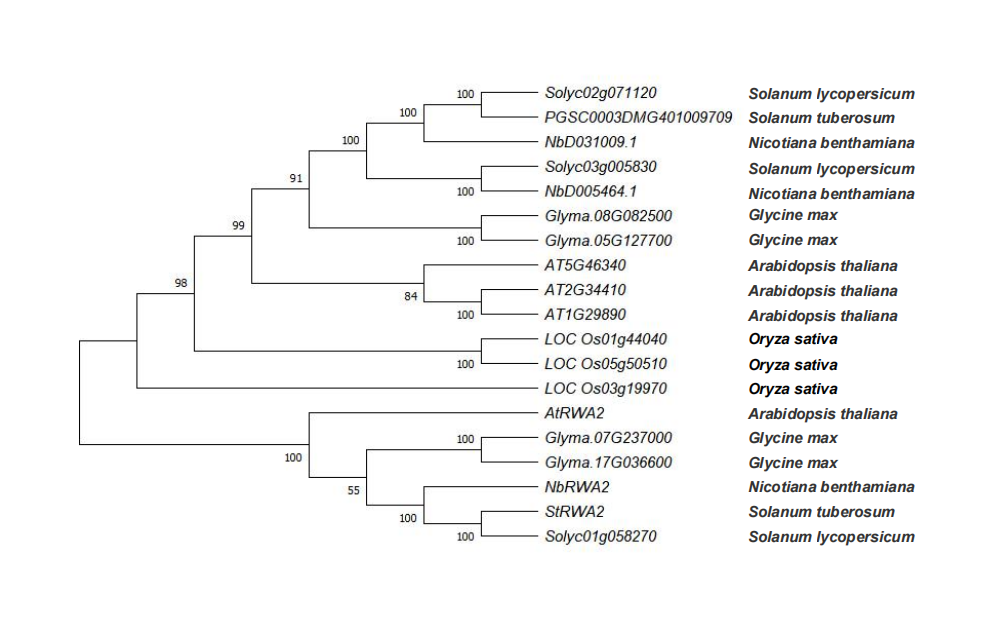


**Fig S1.** Phylogenetic analysis of *StRWA2*. Neighbour-joining phylogenetic tree summarizing the evolutionary relationships of *RWA2* related genes in *Oryza sativa*, *Glycine* *max*, *Arabidopsis thaliana*, *Solanum lycopersicum*, *Nicotiana benthamiana* and *Solanum tuberosum* using the MEGA 7 software. Phylogenetic tree was generated based on a coding sequence (CDS) nucleotide sequence alignment using ClustalW. Bootstrapping was performed with 1000 replicates and values are displayed on branches.


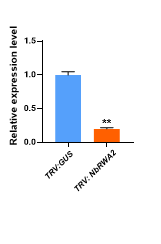


**Fig S2.** Analysis of *NbRWA2* silencing efficiency. The relative expression of *NbRWA2* is tested by qPCR. *TRV: GUS* was used as a control. Error bars indicate the mean ± SEM of three independent biological replicates (***P* < 0.01, Student’s t-test).


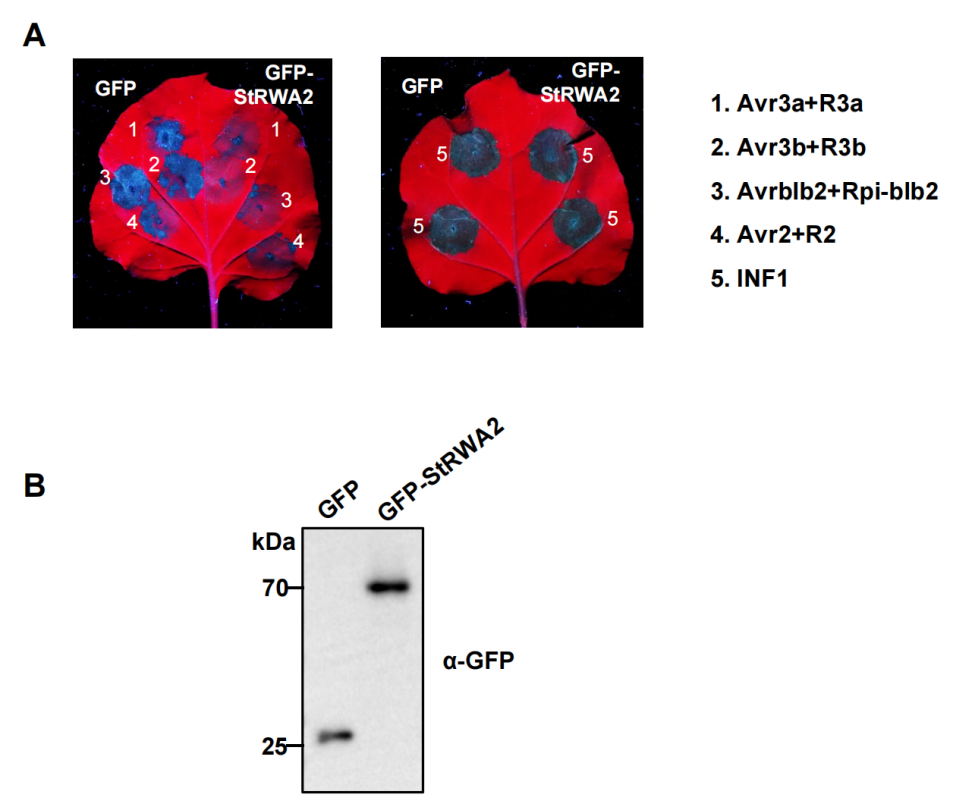


**Fig S3.** StRWA2 inhibits cell death triggered by NLR but not by INF1. (A) Transient expression of GFP-StRWA2 in *N. benthamiana* suppresses R3a/Avr3a-, R3b/Avr3b-, Rpi-blb2/Avrblb2- and R2/Avr2-, but not INF1-triggered cell death. Representative images show the cell death in *N. benthamiana* leaves. After 24 h post-infiltration (hpi) of GFP or GFP-StRWA2a, R3a/Avr3a, R3b/Avr3b, Rpi-blb2/Avrblb2, R2/Avr2 and INF1 were expressed into the same infiltration region. Pictures were taken at 2 days post-infiltration (dpi) under UV. Similar phenotypes were observed in at least three independent experiments. (B) Western blot analysis of StRWA2. GFP or GFP-StRWA2 were expressed in *N. benthamiana* for 48 h and total proteins were incubated with GFP-Trap beads. Proteins were detected by western blotting with anti-GFP antibody.


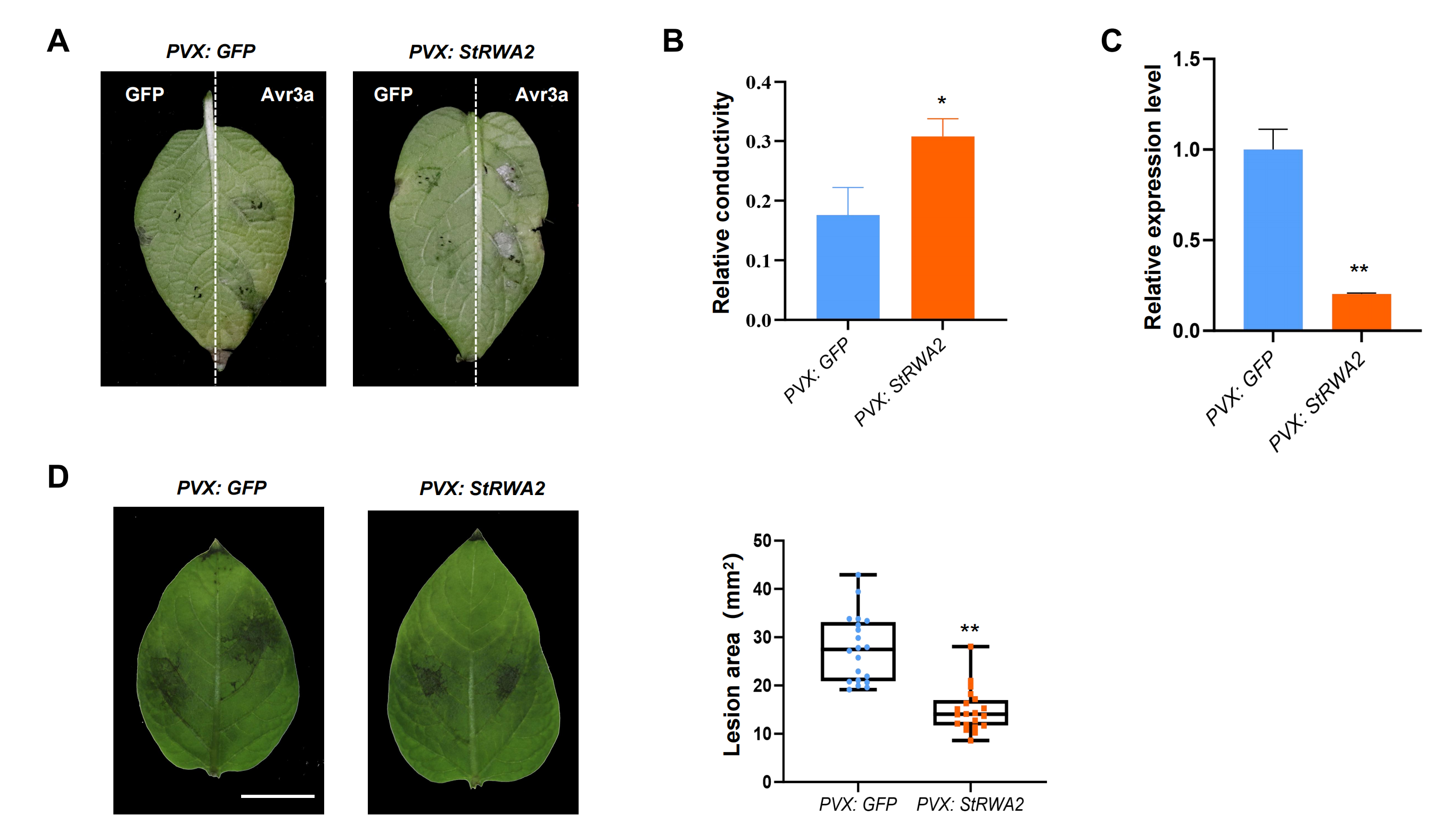


**Fig S4.** (A) Impact of *StRWA2* silencing on resistance to *P. infestans* in *R3a*-containing potato cultivar E10. (A) Earlier and enhanced hypersensitive response (HR) triggered by Avr3a in *StRWA2*-silenced leaves. Representative images of cell death symptoms were captured at 5 days post-infiltration (n = 6). (B) Cell death were quantified by relative electrolyte leakage assay. Quantitative measurement of electrolyte leakage at 72 hours post-infiltration with Avr3a. Error bars indicate ± SDs, n=3 (*P < 0.05, Student’s t-test).

(C) Analysis of *StRWA2* silencing efficiency. The relative expression of *StRWA2* is tested by qPCR. *PVX: GFP* was used as a control. Error bars indicate the mean ± SEM of three independent biological replicates (***P* < 0.01, Student’s t-test). (D) Infection phenotypes of *P. infestans* on *StRWA2*-silenced potato leaves. Images were captured at 3 dpi. Scale bar = 1 cm. Standard deviations are displayed as error bars, n ≥ 15 (**P < 0.01, Student’s t-test).


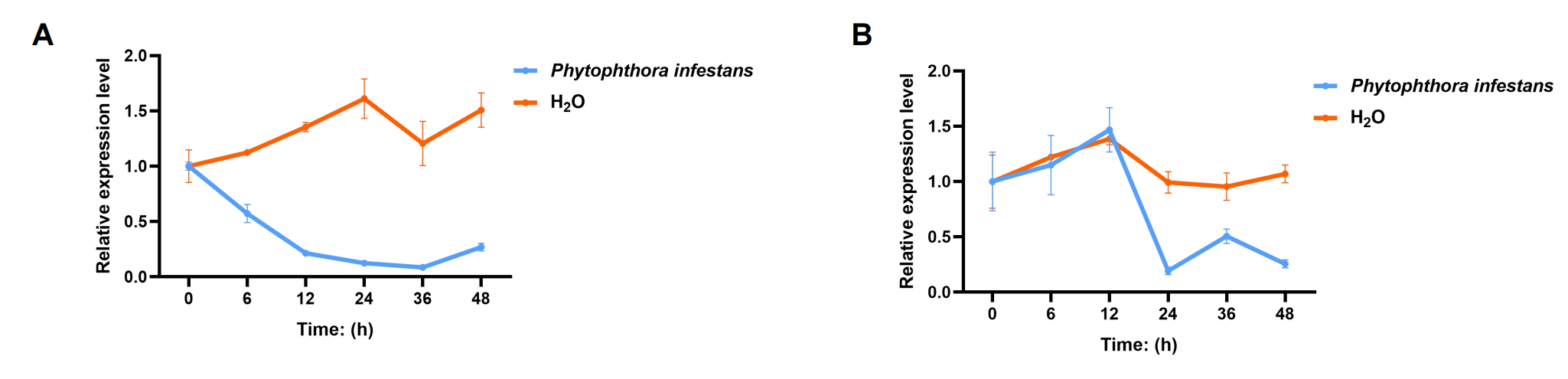


**Fig S5.** Expression pattern of *StRWA2* during *P. infestans* infection. Relative expression levels of *StRWA2* were determined by qPCR in potato cultivars E10 (containing *R3a*) and E3 (without *R3a* or *Rpi-blb2*) at indicated time points (0, 6, 12, 24, 36, and 48 hpi) following inoculation with *P. infestans* or mock treatment (water). (A) In E10, *StRWA2* shows a rapid and continuous down-regulation starting from 6 hpi. (B) In E3, *StRWA2* expression remains stable in the early stages and significantly decreases starting from 24 hpi. Error bars indicate the mean ± SD of three biological replicates.


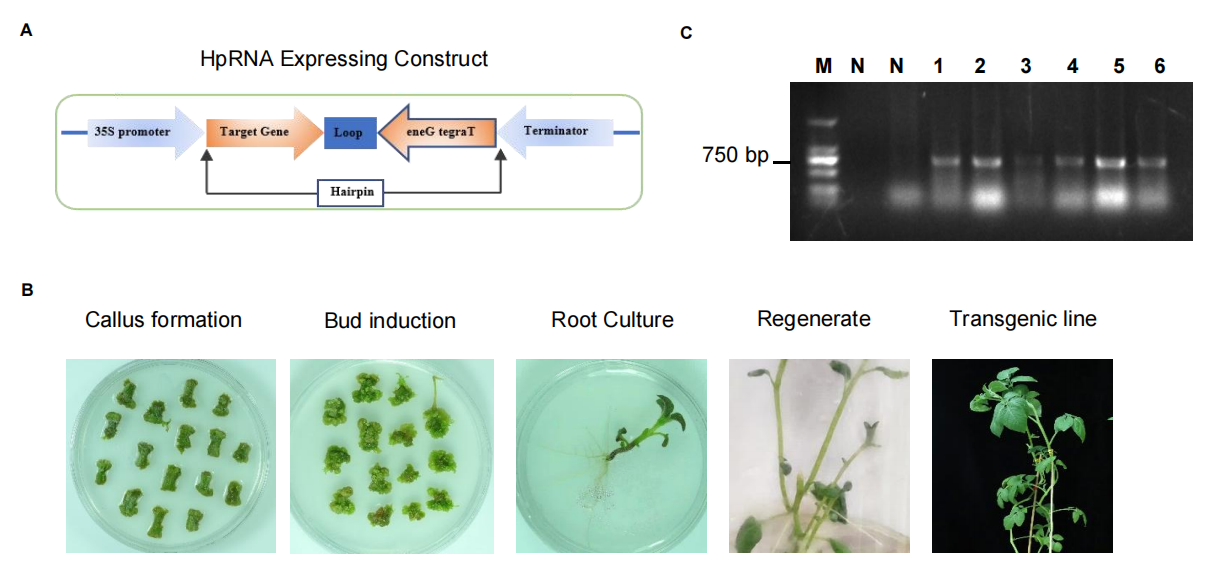


**Fig S6.** Genetic transformation to construct *StRWA2*-silenced potato plants. (A) Construction diagram of RNAi vector pHellsgate8 to form hairpin structure. (B) Representative images showing RNAi potatoes at each stage from callus formation to the whole transgenic plants. (C) More than five independent transformants were obtained and confirmed by PCR using Fw-kan and Rv-kan pair primers (Supplementary Table 1). The size of the amplified kana fragment was 822 bp. N, negative control.


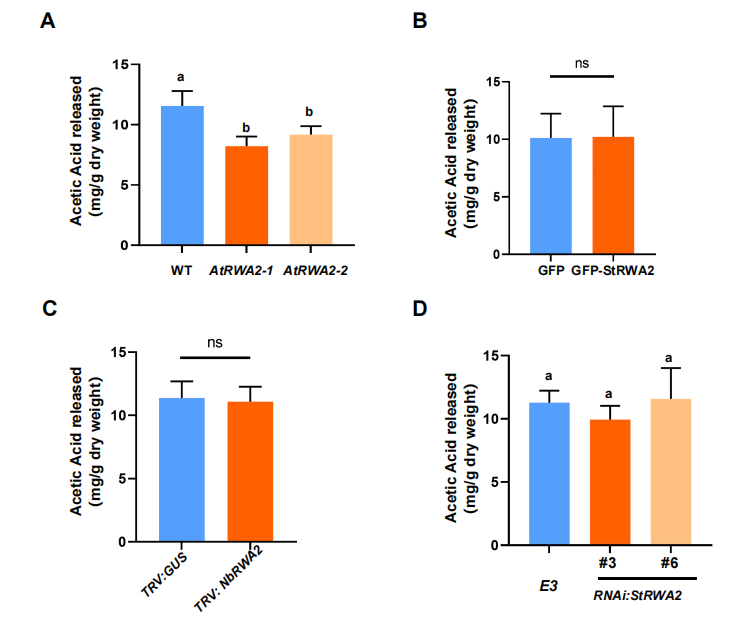


**Fig S7.** Transient overexpression and silencing of *RWA2* orthologs show no significant effect on cell wall acetylation in *N. benthamiana* and *S. tuberosum*. (A) The positive control (*AtRWA2* mutant) exhibited a significant reduction in acetic acid release. Two additional T-DNA insertional mutants of *RWA2*, *AtRWA2-1* (SALK_003787), *AtRWA2-2* (SALK_078630)*.* (B) Transient expression of StRWA2 in *N. benthamiana* did not significantly affect cell wall acetylation. (C, D) No significant difference in acetic acid release between *NbRWA2*- or *StRWA2*-silenced plants and wild-type (WT) plants.


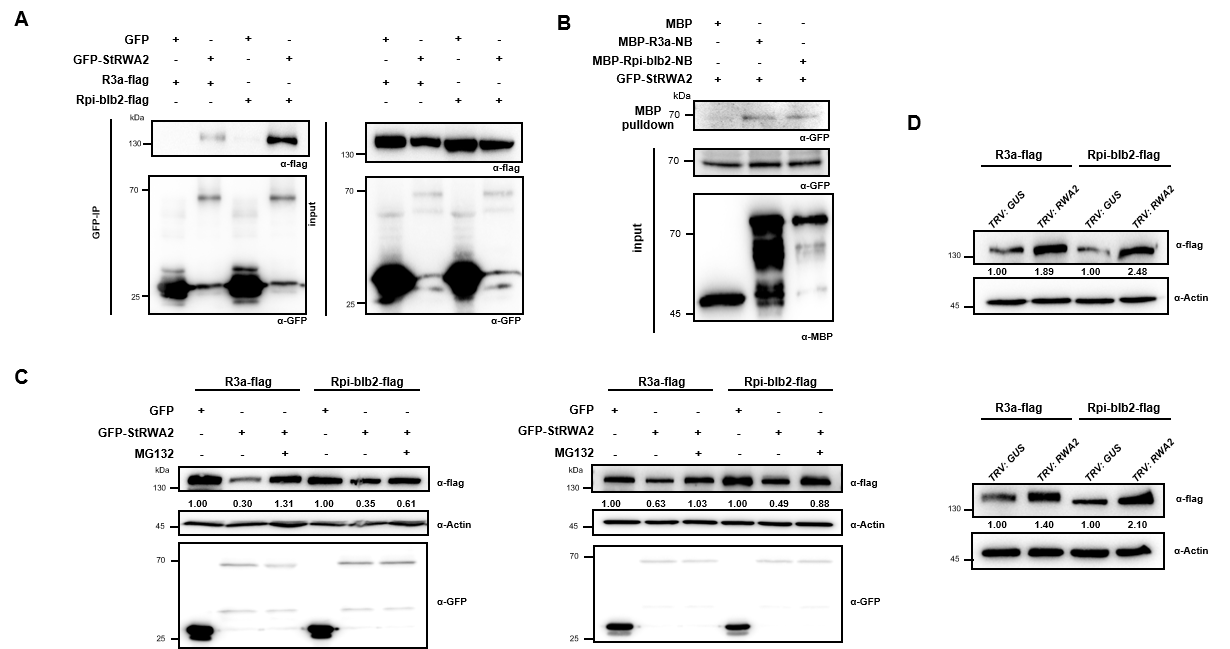


**Fig S8.** Independent immunoblot replicates demonstrate that StRWA2 associates with R3a and Rpi-blb2 and facilitates their degradation via the 26S proteasome pathway. (A, B) Co-IP assay and Semi-*in vivo* pull-down assay confirmed StRWA2 associates with R3a and Rpi-blb2. (C) StRWA2 facilitated R3a and Rpi-blb2 degradation in a 26S proteasome manner. Left and right panels represent two independent biological replicates. (D) Silencing of *NbRWA2* enhanced the accumulation of R3a and Rpi-blb2 in *N. benthamiana*. Upper and lower panels represent two independent biological replicates.


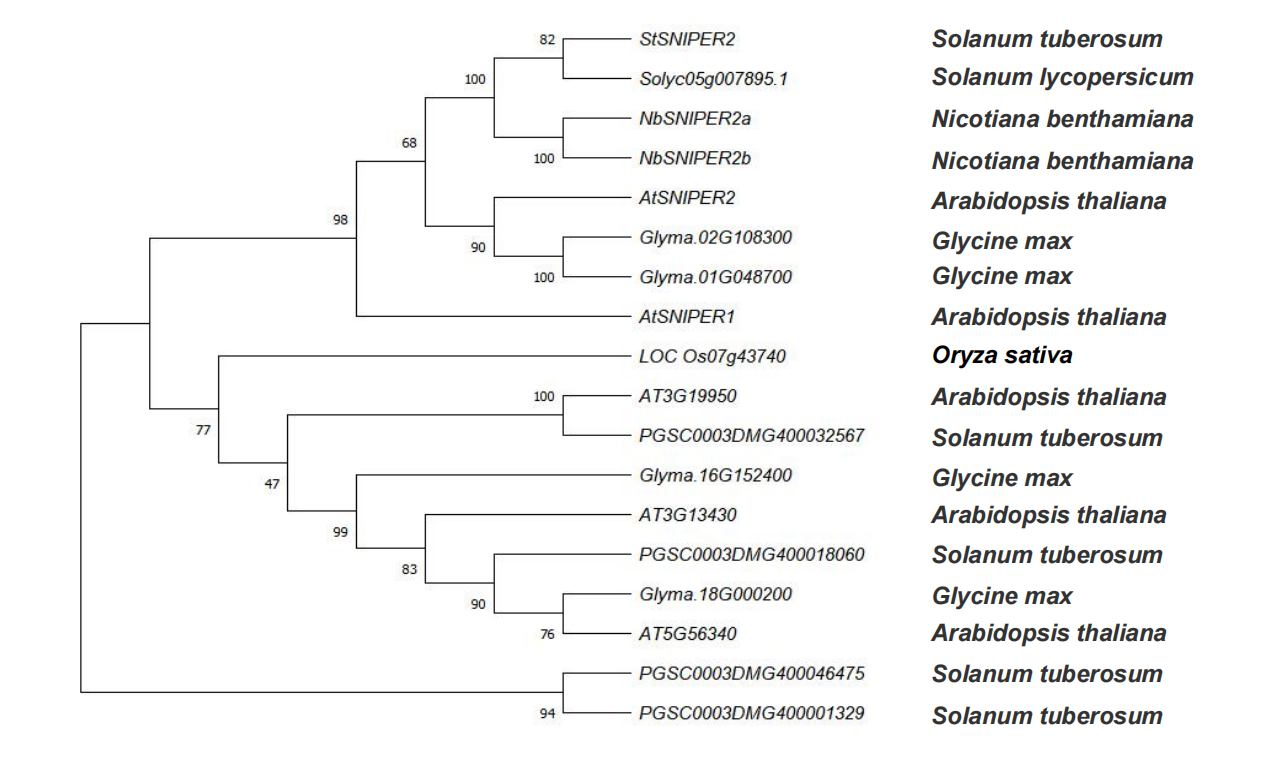


**Fig S9.** Phylogenetic analysis of *StSNIPER2*. Neighbour-joining phylogenetic tree summarizing the evolutionary relationships of *SNIPER2* related genes in *Oryza sativa*, *Glycine* *max*, *Arabidopsis thaliana*, *Solanum lycopersicum*, *Nicotiana benthamiana* and *Solanum tuberosum* using MEGA 7 software. Phylogenetic tree was generated based on a coding sequence (CDS) nucleotide sequence alignment using ClustalW. Bootstrapping was performed with 1000 replicates and values are displayed on branches.


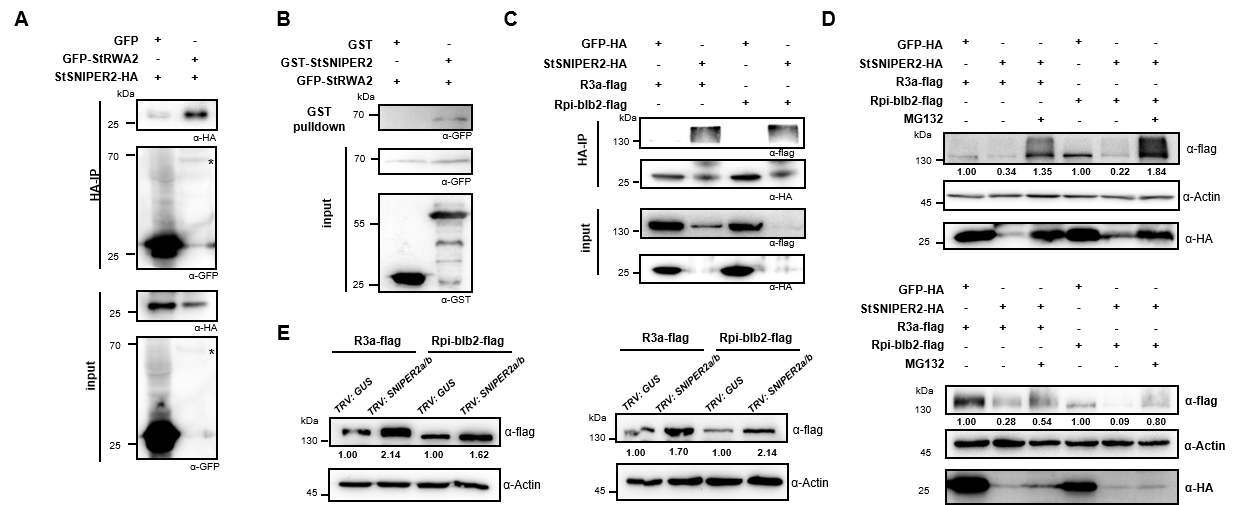


**Fig S10.** Independent immunoblot replicates demonstrate that StSNIPER2 associates with StRWA2 and targets R3a/Rpi-blb2 for degradation. (A) Reciprocal Co-IP assay and Semi-*in vivo* pull-down assay confirmed StRWA2 associates with StSNIPER. (B) Semi-*in vivo* pull-down assay confirmed StRWA2 associates with StSNIPER2. (C) Co-IP assay confirmed StSNIPER associates with R3a/Rpi-blb2. (D) StSNIPER2 facilitated R3a and Rpi-blb2 degradation in a 26S proteasome manner. Upper and lower panels represent two independent biological replicates. (E) Silencing of *NbSNIPER2a/b* enhanced the accumulation of R3a and Rpi-blb2 in *N. benthamiana*. Left and right panels represent two independent biological replicates.


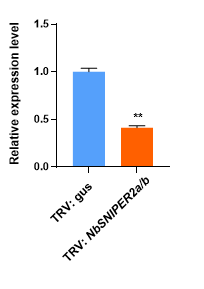


**Fig S11.** Analysis of *NbSNIPER2a/b* silencing efficiency. The relative expression of *NbSNIPER2a/b* is tested by qPCR. *TRV: GUS* was used as a control. Error bars indicate the mean ± SEM of three independent biological replicates (***P* < 0.01, Student’s t-test).


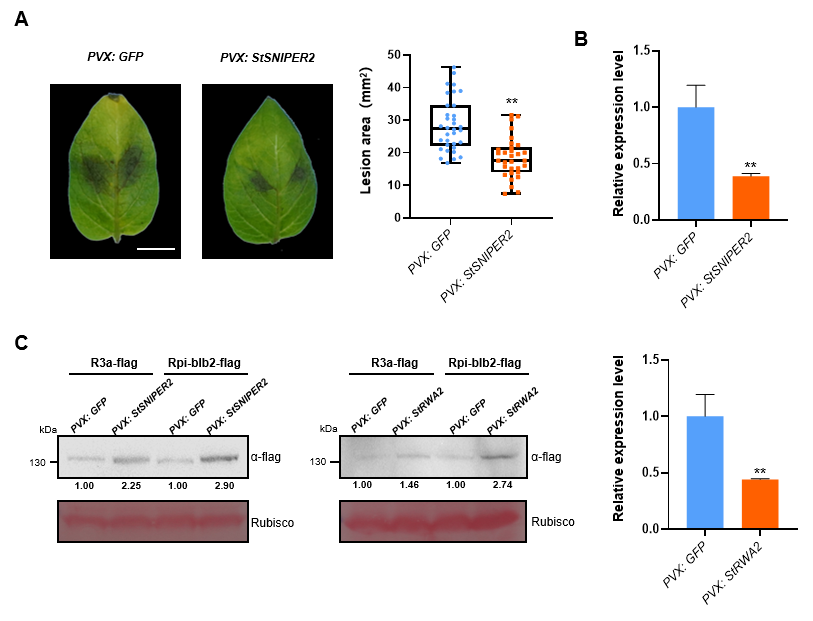


**Fig S12.** *StSNIPER2*-silenced potato plants show enhanced resistance to *P. infestans*. (A) Infection phenotypes of *P. infestans* on *StSNIPER2*-silenced potato leaves. Images were captured at 3 dpi. Scale bar = 1 cm. Standard deviations are displayed as error bars, n ≥ 15 (**P < 0.01, Student’s t-test). (B) Analysis of *StSNIPER2/StRWA2* silencing efficiency. The relative expression of *StSNIPER2/StRWA2* is tested by qPCR. *PVX: GFP* was used as a control. Error bars indicate the mean ± SEM of three independent biological replicates (***P* < 0.01, Student’s t-test). (C) Silencing of *StSNIPER2* or *StRWA2* promotes the accumulation of NLRs in potato. R3a-flag and Rpi-blb2-flag was transiently expressed in *PVX: GFP* and *PVX: StSNIPER2* or *PVX: StRWA2* plants. Rubisco was used to indicates the loading control. Relative Gray values are calculated using IMAGE J. Numbers indicate the relative intensity of R3a or Rpi-blb2 normalized to *PVX: GFP* control.


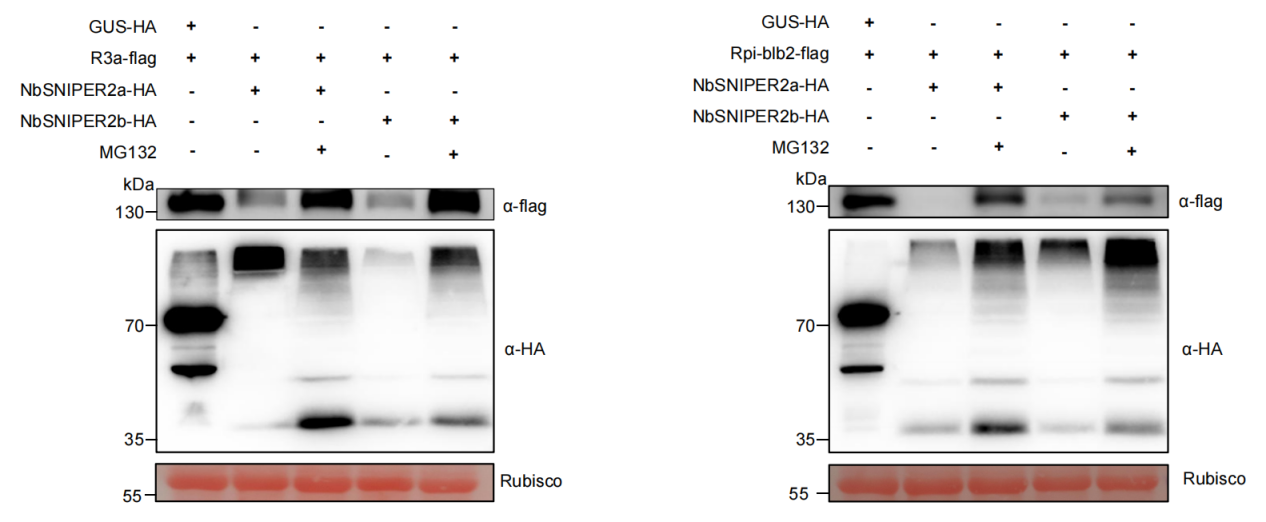


**Fig S13.** NbSNIPER2a/b facilitated R3a and Rpi-blb2 degradation via 26S proteasome. GUS-HA or NbSNIPER2a/b-HA and R3a-flag or Rpi-blb2-flag were transiently expressed in *N. benthamiana*. Leaves were treated with 50 µM MG132 at 40 hpi and proteins were detected by western blotting using anti-flag and anti-HA antibody. Ponceau S staining of Rubisco indicates the loading control.


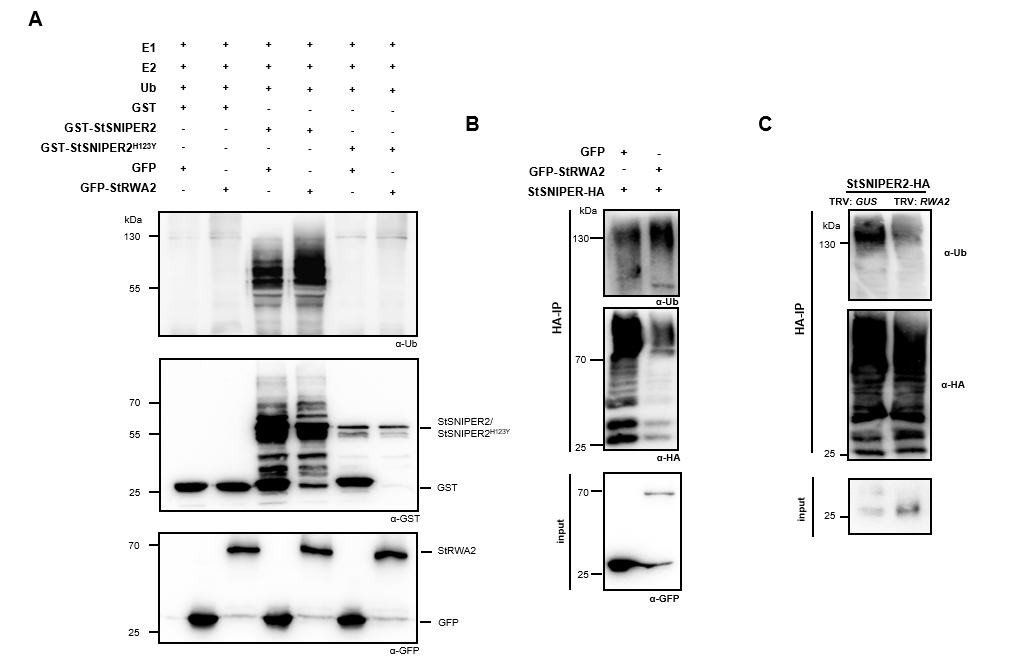


**Fig S14.** RWA2 enhances the auto-ubiquitination of StSNIPER2. (A, B) *Semi-in vitro* ubiquitination assay and *in vivo* ubiquitination assay confirmed the E3 ubiquitin ligase activity of StSNIPER2 is enhanced by StRWA2. For semi-in vitro ubiquitination assay, GFP, GST, and GST-StSNIPER2H123Y served as negative controls. For *in vivo* ubiquitination assay GFP served as negative control. (C) Ubiquitination activity of StSNIPER2 was attenuated in *NbRWA2* silencing plant. StSNIPER2-HA was transiently expressed in *TRV: GUS* or *TRV: NbRWA2* plants, leaves were treated with 50 µM MG132 for 2h and and total proteins were incubated with HA-Trap beads.


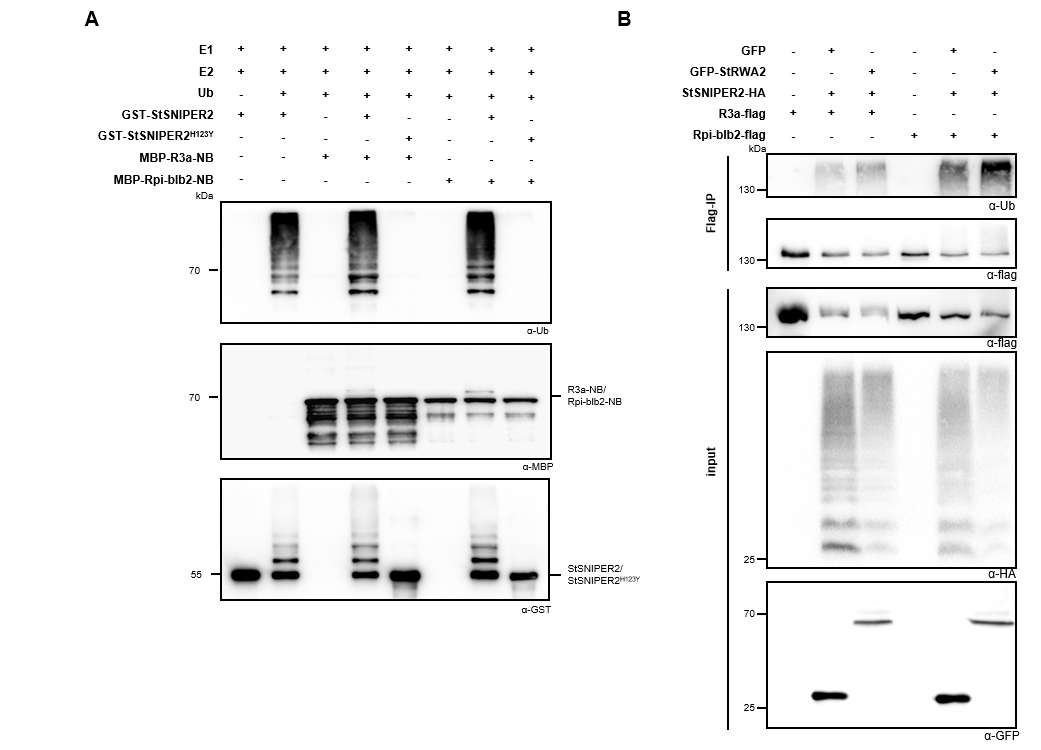


**Fig S15.** Independent immunoblot replicates demonstrate that StRWA2 enhances StSNIPER2-mediated ubiquitination of R3a and Rpi-blb2. (A) *In vitro* ubiquitination assays demonstrated that R3a and Rpi-blb2 are ubiquitinated by StSNIPER2. (B) StRWA2 enhances StSNIPER2-mediated ubiquitination of R3a and Rpi-blb2 *in vivo*.


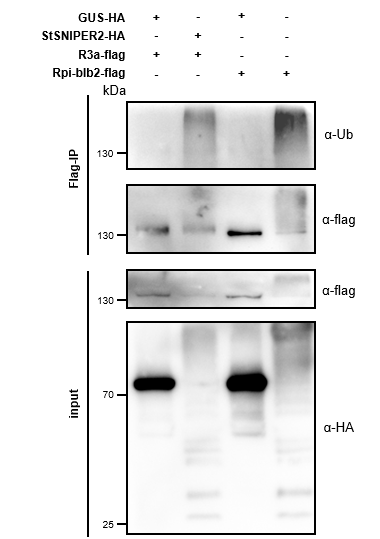


**Fig S16.** *In vivo* ubiquitination assays demonstrated that R3a and Rpi-blb2 are ubiquitinated by StSNIPER2. GUS-HA or StSNIPER2-HA and R3a-flag or Rpi-blb2-flag were transiently expressed in *N. benthamiana*. Leaves were treated with 50 µM MG132 for 2h. Total proteins were incubated with Flag-Trap beads and ubiquitination was detected by western blotting using anti-flag and anti-Ub antibody.


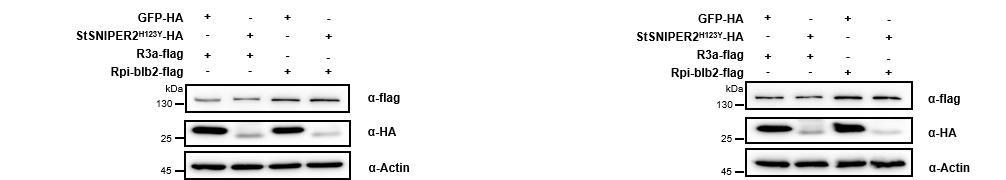


**Fig S17.** Independent immunoblot replicates demonstrate that StSNIPER2^H123Y^ does not affect the stability of R3a or Rpi-blb2. GFP-HA or StSNIPER2^H123Y^-HA and R3a-flag or Rpi-blb2-flag were transiently expressed in *N. benthamiana* and harvested at 48 h. α-actin antibody was used to indicates the loading control. Left and right panels represent two independent biological replicates.


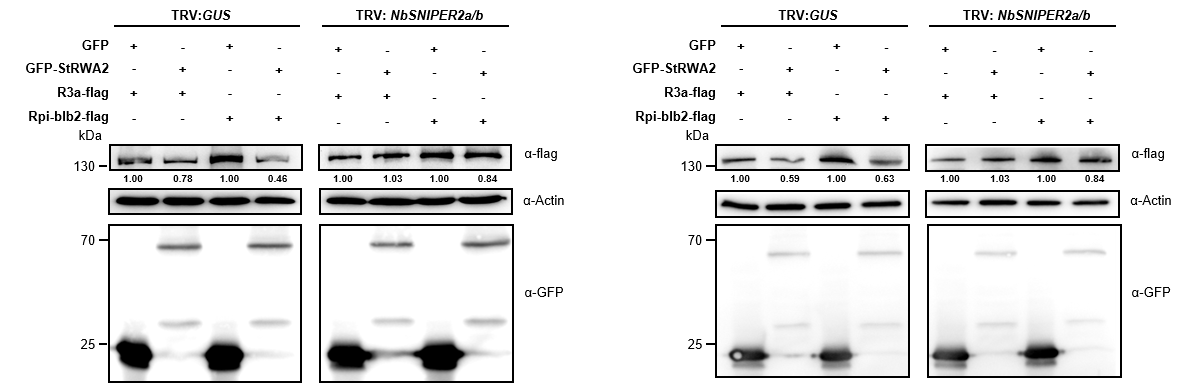


**Fig S18.** Independent immunoblot replicates demonstrate that SNIPER2 is essential for the StRWA2-meditaed destabilization of R3a and Rpi-blb2. GFP or GFP-StRWA2 and R3a-flag or blb2-flag were transiently expressed in *TRV: GUS* and *TRV: NbSNIPER2a/b* plants. Proteins were detected with anti-flag, anti-GFP antibodies and anti-actin antibody was used to indicates the loading control. Relative gray values are calculated using IMAGE J. Numbers indicate the relative intensity of R3a or Rpi-blb2 to plant actin normalized to GFP control. Left and right panels represent two independent biological replicates.

**Table S1. List of primers**

| Primers | Sequence (5'-3') |
| --- | --- |
| R3a-flag-F | GGGGACGAGCTCGGTACCATGGAGATTGGCTTAGCA |
| R3a-flag-R | GTCTTTGTAGTCGTCGACCATGCATTCCCTATCGAT |
| Rpi-blb2-flag-F | GGGGACGAGCTCGGTACCATGGAAAAACGAAAAGAT |
| Rpi-blb2-flag-R | GTCTTTGTAGTCGTCGACCTTAAATAACGGGATATC |
| MBP-R3a-NB-F | CGCGATATCGTCGACGGATCCCAGAATGATATAGAGGAT |
| MBP-R3a-NB-R | TTAATTACCTGCAGGGAATTCTTAGTAGCTCAACATCAA |
| MBP-blb2-NB-F | CGCGATATCGTCGACGGATCCATACTTAGAAAGCTCACC |
| MBP-blb2-NB-R | TTAATTACCTGCAGGGAATTCTTAGTCATAACTTATTTC |
| GST-StSNIPER2 | GATCTGGTTCCGCGTGGATCCATGAATTCCAGAAAC |
| GST-StSNIPER2 | GATGCGGCCGCTCGAGTCGACAGCTTCTGATGCATG |
| StSNIPER2-HA-F | GGGGACGAGCTCGGTACCATGAATTCCAGAAACATA |
| StSNIPER2-HA-R | AACATCGTATGGGTAGTCGACAGCTTCTGATGCATGATC |
| StSNIPER2H123Y-HA-R1 | TCCATGAAATCTATACTTACAAGGCATTTCTT |
| StSNIPER2H123Y-HA-F2 | GCCTTGTAAGTATAGATTTCATGGAGATTG |
| PVX-SNIPER2-F | TCAGCACCAGCTAGCATCGATATGAATTCCAGAAACATAGACCTTTCT |
| PVX-SNIPER2-R | AGAGGATCCCCGGGAATCGATTTCACTACCCCACCAATTACCC |
| PVX-RWA2-F | TCAGCACCAGCTAGCATCGATATGCTAATCTACGGCCCCCT |
| PVX-RWA2-R | AGAGGATCCCCGGGAATCGATGATGAAAAGATCACGATTGTAGCTCT |
| NbSNIPER2a-HA-F | GGGGACGAGCTCGGTACCATGGCTTCAGAATCAGAATC |
| NbSNIPER2a-HA-R | AACATCGTATGGGTAGTCGACAAGCTCTGGTTCATGATCAT |
| NbSNIPER2b-HA-F | GGGGACGAGCTCGGTACCATGGCTTCAGAATCAGAAAG |
| NbSNIPER2b-HA-R | AACATCGTATGGGTAGTCGACAACCTCTGGTTGATGATCAT |
| TRV-NbRWA2-F | GTGAGTAAGGTTACCGAATTCCGTGATCTTTTCCTCTT |
| TRV-NbRWA2-R | GAGACGCGTGAGCTCGGTACCCTGAGTGAATCTTGCAAT |
| TRV-NbSNIPER2-F | GTGAGTAAGGTTACCGAATTCTTCGAATTTTGAGTC |
| TRV-NbSNIPER2-R | GAGACGCGTGAGCTCGGTACCCCTCTATTTTCACTC |
| RT-NbSNIPER2a-F | GAGGCATCATCATTCA |
| RT-NbSNIPER2a-R | GTTGATCTGGGTCTGT |
| RT-NbSNIPER2b-F | GGAGGCATCGTCATT |
| RT-NbSNIPER2b-R | ATCTGGGTCGGTTGA |
| RT-NbRWA2-F | CTATTGGAAGGAGGTG |
| RT-NbRWA2-R | TACGGTCAGATAGGTAAA |
| RNAi-RWA2-F1 | TTTGGAGAGGACACGCTCGAGTACAAGAAAAATTCAGCATCATCCA |
| RNAi-RWA2-R1 | TCCTTACCAAGCTGGGGTACCTTTCAAGCAAGAAAGTTTCATCCA |
| RNAi-RWA2-F2 | GAAATTGGGTTCGAAATCGATTTTCAAGCAAGAAAGTTTCATCCA |
| RNAi-RWA2-R2 | TCATTAAAGCAGGACTCTAGATACAAGAAAAATTCAGCATCATCCA |
| RT-StRWA2-F | AGGAGGTGGTCTTCAA |
| RT-StRWA2-R | TACGGTCAGATAGGTAAA |
| RT-StEFla-F | ATTGGAAACGGATATGCTCCA |
| RT-StEFla-R | TCCTTACCTGAACGCCTGTCA |
| RT-StPR1-F | TGGTGATTTCACGGGGAGGG |
| RT-StPR1-R | CGAACTGAGTTGCGCCAGAC |
| RT-StWRKY1-F | GAAGAATAAAGCCGGGTTCTTGG |
| RT-StWRKY1-R | CTTACACGATTTGATCACCTCATCC |
| RT-NbEF1α-F | TGGACACAGGGACTTCATCA |
| RT-NbEF1α-R | CAAGGGTGAAAGCAAGCAAT |
| RT-NbWRKY7-F | CACAAGGGTACAAACAACACAG |
| RT-NbWRKY7-R | GGTTGCATTTGGTTCATGTAAG |
| RT-NbPR1-F | ATGGTCAATACGGCGAAAAC |
| RT-NbPR1-R | CCTAGCACATCCAACACGAA |
| KanR-F | ATGGCAATTACCTTATCCGCAA |
| KanR-R | TCAGAAGAACTCGTCAAGAAGG |
